# The relationship between pan immune inflammatory index (PIV) and 28 day mortality in patients with severe urinary sepsis: a retrospective study of a multinational dual cohort study

**DOI:** 10.3389/fmed.2026.1802352

**Published:** 2026-07-10

**Authors:** Wenjun Zhang, Kunyuan Huang, Xiaobo Li, Guosheng Chen, Chengjia Wang, Kun Yang, Kaifa Tang

**Affiliations:** 1 Department of Urology, Anshun People’s Hospital, Anshun, China; 2 Department of Urology, The First Affiliated Hospital of Guizhou University of Traditional Chinese Medicine, Guiyang, China; 3 Department of Urology, Xingyi People’s Hospital, Xingyi, China

**Keywords:** biomarkers, pan inflammatory immune markers (PIV), poor prognosis, risk stratification, urinary sepsis

## Abstract

**Background:**

Urosepsis is a critical condition originating from urinary tract infections, characterized by rapid progression and high mortality. The Pan-immune-inflammation Value (PIV), a novel composite index reflecting integrated immune and inflammatory status, has shown prognostic value in various critically ill and septic populations. However, its independent prognostic value in urosepsis remains inadequately explored. This study therefore aims to systematically evaluate the ability of PIV to predict 28-day adverse outcomes in patients with urosepsis.

**Materials and methods:**

This study employed a retrospective dual-cohort design. The internal training cohort comprised adult ICU patients with urosepsis from the MIMIC-IV database, while the external validation cohort was derived from the electronic medical record system of Anshun Municipal People’s Hospital. To investigate the association between the PIV and short-term adverse outcomes in urosepsis patients, we utilized a range of statistical methods, including multivariable Cox regression, restricted cubic spline (RCS) analysis, subgroup analysis, and Kaplan-Meier survival curves. To develop a parsimonious predictive model, the internal cohort was randomly split into training and testing sets at a 7:3 ratio. Within the training set, an ensemble machine learning strategy—incorporating the Boruta algorithm, LASSO-Cox regression, random forest (RF), gradient boosting (GBDT), and support vector machine (SVM)—was applied to identify key predictive variables from serological tests, comorbidities, demographic characteristics, and vital signs. Based on the selected features, prognostic models were constructed using multivariable Cox regression in the training, testing, and external validation sets, respectively. The discriminatory power of these models against traditional disease severity scores was assessed using receiver operating characteristic (ROC) curves, with the area under the curve (AUC) quantifying predictive performance.

**Result:**

A total of 1,686 patients with severe urosepsis were included in this study. In the fully adjusted model, both continuous PIV and PIV quartiles were independently associated with 28-day adverse outcomes. For 28-day ICU mortality, each unit increase in continuous PIV was associated with a 76.4% higher risk (HR 1.764, 95% CI 1.340–2.323, *p* < 0.001); compared with the lowest quartile (Q1), the highest quartile (Q4) showed a significantly increased risk of 84.3% (HR 1.843, 95% CI 1.186–2.846, *p* = 0.007). For 28-day in-hospital mortality, the HR for continuous PIV was 1.639 (95% CI 1.214–2.214, *p* < 0.001), and the HR for Q4 was 1.789 (95% CI 1.110–2.885, *p* = 0.039). In the external validation cohort, the predictive value of PIV for 28-day ICU mortality remained consistent and was even more pronounced (continuous PIV: HR 1.79, 95% CI 1.21–2.64, *p* = 0.004). Restricted cubic spline analysis further confirmed a significant positive dose-response relationship between PIV and mortality (overall *p* < 0.001). The risk prediction model based on PIV demonstrated good discriminative ability, with areas under the receiver operating characteristic curve of 0.71, 0.73, and 0.76 in the training, internal testing, and external validation sets, respectively, all of which were higher than those of traditional severity scores (e.g., SOFA, APACHE II).

**Conclusion:**

This study confirms PIV as an independent predictor of 28-day mortality risk in patients with urosepsis across two cohorts. The prediction model incorporating PIV and four other clinical variables exhibited good discrimination and calibration, with prognostic performance superior to that of traditional disease severity scores. Future prospective, multicenter studies involving diverse geographic and ethnic populations are needed to further validate the generalizability of this model, thereby facilitating precise risk stratification for patients with urosepsis.

## Introduction

Sepsis is defined as life-threatening organ dysfunction caused by a dysregulated host response to infection and is a leading cause of mortality in non-cardiac intensive care units (ICUs) worldwide. It affects millions annually and imposes a substantial healthcare burden (1, 2). Urosepsis represents a major clinical subtype, defined as a critical systemic infection originating from the urinary tract, such as pyelonephritis or complicated urinary tract infection. It accounts for approximately 9–31% of all sepsis cases, depending on geographic region and the study population (3, 4). These infections are predominantly caused by Gram-negative bacilli like *Escherichia coli* and Klebsiella species. The endotoxins released by these pathogens can trigger a potent systemic inflammatory cascade, leading to endothelial injury and microcirculatory dysfunction (5, 6). Notably, challenges in managing urosepsis are intensifying due to an aging population, increasing rates of urinary obstructive diseases, and the growing threat of antibiotic resistance. Patients frequently face risks of shock, acute kidney injury, and other organ failures, resulting in persistently high rates of mortality and morbidity (7, 8). Consequently, identifying biomarkers capable of early and objective reflection of its pathophysiological disturbance and prognosis is crucial for enabling precise risk stratification and timely intervention.

Current clinical assessment and prognostic evaluation of urosepsis primarily rely on two categories of tools: comprehensive scoring systems, such as the Sequential Organ Failure Assessment (SOFA) and Acute Physiology and Chronic Health Evaluation (APACHE II) scores, which integrate multiple physiological parameters; and traditional inflammatory biomarkers, including C-reactive protein (CRP) and procalcitonin (PCT) (9, 10). However, these methods have inherent limitations. Scoring systems are often cumbersome due to their numerous variables and complex calculations. Their results can be confounded by therapeutic interventions like sedation and mechanical ventilation, and they lack convenience for dynamic monitoring. Although PCT and CRP are indicative of bacterial infection, their levels are influenced by factors such as infection site, renal function, and non-infectious inflammatory states, resulting in limited specificity. Moreover, they fail to comprehensively reflect the complex state of the host’s immune-inflammatory network (11, 12). Consequently, PIV represents one promising integrative marker among several emerging tools that can combine multidimensional immune-inflammatory information, is easily obtainable, and demonstrates high stability.

In recent years, the PIV, a composite hematological index, has garnered increasing attention. PIV integrates key cellular components—neutrophils and monocytes (reflecting innate immune activation), platelets (involved in inflammation and thrombosis), and lymphocytes (representing immunoregulatory function)—into a single metric. This allows for an assessment of systemic inflammatory intensity, immune response status, and immunothrombotic tendency (13, 14). A growing body of evidence demonstrates that PIV holds superior prognostic value compared to individual blood cell parameters across various solid tumors, cardiovascular diseases, and infectious conditions, underscoring its role as a broadly relevant integrative indicator of pathophysiology (15–17). Mechanistically, the progression of urosepsis is frequently characterized by neutrophil hyperactivation, lymphocyte depletion, monocyte dysfunction, and platelet activation contributing to microthrombosis—precisely the pathophysiological shifts that PIV reflects. Consequently, within the urosepsis population, PIV holds theoretical promise as a sensitive and holistic biomarker of the core pathological cascade, encompassing excessive inflammation, immune suppression, and endothelial injury, positioning it as a promising and easily accessible tool for prognostic assessment.

However, despite growing evidence for the prognostic value of PIV in sepsis, its independent association with short-term outcomes in patients with urosepsis has not been systematically evaluated. There is a notable lack of high-quality clinical studies specifically investigating the association of PIV with short-term adverse outcomes (such as 28-day mortality and progression of organ failure) in urosepsis patients, its independent predictive performance, and its comparative advantage over traditional scoring systems. Therefore, this study aims to systematically investigate the association between admission PIV levels and short-term adverse outcomes in patients with urosepsis through a retrospective dual-cohort analysis, and to compare its prognostic performance with established disease-severity scores. We utilized the large public critical care database (MIMIC-IV, version 2.2) to form the training and internal validation cohorts, and collected independent data from a regional medical center for external validation. Various statistical methods, including multivariable Cox regression, restricted cubic spline analysis, survival analysis, and construction of a nomogram prediction model, were employed to comprehensively evaluate the independent prognostic value of PIV. Ultimately, we aim to develop a simple and effective quantitative tool.

## Materials and methods

### Data source

This study employed a retrospective dual-cohort design to enhance the robustness and generalizability of the findings. The internal cohort data were sourced from the Medical Information Mart for Intensive Care IV (MIMIC-IV, v2.2), an international public critical care database. This repository contains de-identified, high*quality data from more than 50,000 unique ICU patients admitted to the Beth Israel Deaconess Medical Center between 2008 and 2019. The creation of this database received approval from the relevant Institutional Review Boards (IRB protocol numbers: 2001P001699, 0403000206), and all patient data were de-identified to protect privacy (18). To further validate the general applicability of our results across different institutions and populations, we established an external validation cohort. This cohort comprised patients with urosepsis admitted to Anshun Municipal People’s Hospital between January 2022 and December 2025. The study protocol was reviewed and approved by the Ethics Committee of Anshun Municipal People’s Hospital.

### Study population

This study focused on patients admitted to the intensive care unit (ICU) with urosepsis. Sepsis was diagnosed according to the international Sepsis-3 consensus criteria, defined as an increase in the Sequential Organ Failure Assessment (SOFA) score by ≥2 points from baseline. Urinary tract infection (UTI) was ascertained differently in the two cohorts. In the internal cohort, UTI was identified based on International Classification of Diseases (ICD) diagnostic codes within the MIMIC-IV database. UTI was ascertained differently in the two cohorts. In the internal cohort, UTI was identified based on the following ICD-9 and ICD-10 diagnostic codes within the MIMIC-IV database: ICD-9 codes 5990, 590.1, 590.8, 595; and ICD-10 codes N39.0, N10, N12, N30.0, N30.9. In the external validation cohort, diagnosis relied on a combination of typical clinical presentations (urinalysis indicating pyuria and/or bacteriuria) and positive urine culture results. Only patients with an initial admission diagnosis of UTI who simultaneously met the Sepsis-3 criteria were included in the urosepsis cohort. All enrolled patients met the following criteria: (1) age ≥ 18 years; (2) hospital and ICU stay lasting at least 24 h; (3) availability of complete records for serum lactate and albumin levels at ICU admission; (4) exclusion of patients with malignancy due to concern that tumor-related leukocytosis, thrombocytosis, or lymphopenia could confound the PIV measurement independently of infection, thereby altering the association between PIV and urosepsis outcomes.

### Variable extraction

Data extraction was performed using PostgreSQL (v13.7.2) and Navicat Premium (v16) software by executing structured query language (SQL). The extracted variables were categorized into six groups: (1) demographics; (2) comorbidities; (3) vital signs; (4) disease severity scores at admission; (5) the first laboratory test results within 24 h of admission; and (6) therapeutic interventions. Detailed information for all variables is presented in the baseline characteristics table (Table 1). In the internal MIMIC-IV cohort, we first calculated missingness percentages for all variables. Variables with > 30% missing values were excluded from the analysis. For the remaining variables with < 30% missingness, we assumed data were missing at random (MAR) and performed multiple imputation using the “mice” package in R (version 3.14.0). Specifically, we generated 20 imputed datasets with 20 iterations per imputation. The imputation model included all covariates used in the primary analysis plus the outcome variables (28-day ICU and in-hospital mortality) to reduce bias. Convergence was assessed visually by trace plots of the mean and variance of imputed values across iterations; no systematic trends were observed, and the potential scale reduction factor (PSRF) for all parameters was < 1.1. The imputed datasets were combined using Rubin’s rules. Notably, the four cellular components of PIV (lymphocyte, neutrophil, platelet, and monocyte counts) had 0% missingness, so the primary exposure variable did not require imputation. A complete missing data table (Supplementary Table 7) is provided in the Supplementary materials.

**TABLE 1:** Baseline characteristics of the internal cohort patients with urinary-sepsis, stratified by pan PIV quartiles.

Variables	[ALL]	Q1	Q2	Q3	Q4	*P*-value
	*N* = 1,686	*N* = 421	*N* = 421	*N* = 421	*N* = 423	
logPIV	2.97 (0.60)	2.19 (0.40)	2.81 (0.11)	3.16 (0.10)	3.70 (0.27)	<0.001
Age	69.6 (15.6)	68.2 (15.4)	70.2 (15.2)	69.5 (15.8)	70.4 (16.1)	0.139
Gender						0.812
F	997 (59.1%)	257 (61.0%)	249 (59.1%)	245 (58.2%)	246 (58.2%)	
M	689 (40.9%)	164 (39.0%)	172 (40.9%)	176 (41.8%)	177 (41.8%)	
Race						0.400
Other races	703 (41.7%)	169 (40.1%)	185 (43.9%)	165 (39.2%)	184 (43.5%)	
WHITE	983 (58.3%)	252 (59.9%)	236 (56.1%)	256 (60.8%)	239 (56.5%)	
Weight	79.9 (24.4)	77.9 (22.4)	81.1 (24.9)	81.4 (25.2)	79.2 (24.9)	0.105
hypertension						0.618
No	1037 (61.5%)	253 (60.1%)	254 (60.3%)	259 (61.5%)	271 (64.1%)	
Yes	649 (38.5%)	168 (39.9%)	167 (39.7%)	162 (38.5%)	152 (35.9%)	
AKI:						0.001
No	839 (49.8%)	228 (54.2%)	224 (53.2%)	210 (49.9%)	177 (41.8%)	
Yes	847 (50.2%)	193 (45.8%)	197 (46.8%)	211 (50.1%)	246 (58.2%)	
CKD						
No	1280 (75.9%)	334 (79.3%)	320 (76.0%)	327 (77.7%)	299 (70.7%)	
Yes	406 (24.1%)	87 (20.7%)	101 (24.0%)	94 (22.3%)	124 (29.3%)	0.021
DM:						
No	1066 (63.2%)	263 (62.5%)	259 (61.5%)	269 (63.9%)	275 (65.0%)	
Yes	620 (36.8%)	158 (37.5%)	162 (38.5%)	152 (36.1%)	148 (35.0%)	0.731
HLD						
No	957 (56.8%)	233 (55.3%)	223 (53.0%)	249 (59.1%)	252 (59.6%)	
Yes	729 (43.2%)	188 (44.7%)	198 (47.0%)	172 (40.9%)	171 (40.4%)	0.161
HF						
No	1139 (67.6%)	309 (73.4%)	286 (67.9%)	282 (67.0%)	262 (61.9%)	
Yes	547 (32.4%)	112 (26.6%)	135 (32.1%)	139 (33.0%)	161 (38.1%)	0.005
IHD:						
No	1045 (62.0%)	240 (57.0%)	255 (60.6%)	288 (68.4%)	262 (61.9%)	
Yes	641 (38.0%)	181 (43.0%)	166 (39.4%)	133 (31.6%)	161 (38.1%)	0.007
COPD						
No	1442 (85.5%)	371 (88.1%)	362 (86.0%)	359 (85.3%)	350 (82.7%)	
Yes	244 (14.5%)	50 (11.9%)	59 (14.0%)	62 (14.7%)	73 (17.3%)	0.169
SOFA	6.13 (3.44)	6.50 (3.48)	6.09 (3.16)	5.96 (3.50)	5.99 (3.59)	
APSIII	53.0 (19.7)	52.3 (20.7)	51.8 (19.1)	52.8 (18.7)	55.0 (20.3)	
SAPSII	41.6 (12.9)	41.5 (13.4)	40.9 (13.0)	42.1 (12.5)	41.9 (12.8)	0.018
OASIS	33.6 (8.25)	32.4 (8.17)	33.0 (8.09)	33.7 (8.24)	35.1 (8.29)	< 0.001
CCI	5.75 (2.85)	5.56 (2.83)	5.77 (2.78)	5.70 (3.06)	5.98 (2.72)	< 0.001
APACHEII	19.7 (7.21)	20.2 (7.22)	18.7 (6.63)	18.7 (7.09)	21.1 (7.59)	< 0.001
HR	89.9 (20.4)	86.4 (18.7)	88.8 (20.4)	91.6 (21.2)	92.9 (20.8)	0.047
NBPS	122 (24.9)	119 (24.0)	124 (25.0)	122 (25.8)	122 (24.5)	< 0.001
NBPD	70.0 (19.1)	67.9 (17.7)	71.0 (18.2)	71.3 (19.5)	70.0 (20.9)	< 0.001
NBPM	83.8 (19.2)	81.4 (17.9)	85.0 (18.4)	84.7 (20.2)	83.9 (20.3)	0.035
RR	19.9 (6.29)	18.7 (6.46)	19.6 (6.34)	20.0 (5.44)	21.3 (6.59)	0.027
SpO_2_	96.9 (3.80)	97.3 (3.81)	96.8 (3.70)	96.8 (3.57)	96.4 (4.07)	0.019
Temperaturef	98.0 (5.01)	97.7 (5.26)	98.2 (3.13)	98.2 (4.51)	97.8 (6.52)	< 0.001
lymphocyte_count	1.40 (1.34)	1.64 (1.90)	1.49 (0.95)	1.42 (1.42)	1.05 (0.70)	0.011
HCT	31.8 (6.86)	29.5 (6.50)	32.1 (6.75)	32.8 (6.73)	32.5 (6.95)	0.366
Hb	10.2 (2.27)	9.56 (2.14)	10.4 (2.23)	10.6 (2.23)	10.4 (2.36)	< 0.001
PLT	197 (99.5)	133 (73.2)	182 (73.6)	217 (96.2)	253 (108)	< 0.001
RDW	15.3 (2.66)	15.3 (2.82)	15.3 (2.68)	15.0 (2.40)	15.6 (2.71)	< 0.001
RBC	3.47 (0.81)	3.17 (0.75)	3.53 (0.81)	3.60 (0.76)	3.57 (0.83)	< 0.001
WBC	13.4 (8.19)	9.41 (5.66)	11.6 (4.97)	13.7 (6.74)	18.8 (10.8)	0.008
neutrophil_count	10.7 (7.28)	5.86 (3.85)	8.39 (3.78)	11.0 (5.44)	17.4 (8.94)	< 0.001
ALB	3.02 (0.61)	3.03 (0.59)	3.05 (0.61)	3.07 (0.62)	2.94 (0.62)	< 0.001
AG	14.9 (4.50)	13.9 (4.64)	14.4 (3.91)	15.1 (4.33)	16.1 (4.79)	< 0.001
TCa	8.40 (0.84)	8.33 (0.82)	8.39 (0.90)	8.48 (0.86)	8.40 (0.77)	0.018
Cl	103 (7.48)	105 (7.37)	104 (7.81)	103 (7.20)	102 (7.26)	< 0.001
Glu	152 (79.9)	138 (67.2)	149 (81.3)	154 (81.8)	165 (85.7)	0.078
K	4.22 (0.76)	4.24 (0.80)	4.17 (0.72)	4.19 (0.72)	4.28 (0.78)	< 0.001
Na	138 (6.40)	139 (6.49)	139 (6.71)	139 (6.15)	138 (6.19)	< 0.001
TCO_2_	24.4 (5.69)	25.0 (5.37)	24.6 (5.70)	24.5 (5.59)	23.4 (6.00)	0.132
Lac	2.23 (1.76)	2.27 (1.88)	2.12 (1.59)	2.15 (1.76)	2.38 (1.81)	0.026
PCO_2_	41.7 (11.0)	41.5 (9.99)	41.8 (10.8)	41.8 (11.2)	41.7 (11.7)	0.001
PH	7.37 (0.09)	7.38 (0.09)	7.37 (0.08)	7.37 (0.10)	7.35 (0.10)	0.104
PO_2_	127 (106)	166 (131)	128 (107)	113 (89.8)	99.7 (73.0)	0.977
INR	1.50 (0.81)	1.54 (0.87)	1.45 (0.68)	1.45 (0.66)	1.55 (0.98)	< 0.001
PT	16.4 (9.17)	16.8 (9.56)	15.8 (7.45)	15.8 (7.36)	17.0 (11.6)	< 0.001
APTT	37.8 (23.6)	38.0 (23.3)	36.8 (22.4)	37.0 (23.2)	39.4 (25.2)	0.136
ALT	116 (614)	129 (624)	126 (644)	90.0 (252)	119 (799)	0.131
AST	201 (1090)	213 (1159)	230 (1279)	175 (824)	188 (1052)	0.400
TB	1.79 (4.68)	2.01 (4.28)	1.39 (3.21)	1.70 (4.81)	2.06 (5.96)	0.470
CRE	1.49 (1.38)	1.41 (1.36)	1.40 (1.33)	1.39 (1.17)	1.75 (1.61)	0.882
BUN	30.0 (25.0)	27.4 (23.5)	27.4 (23.4)	29.4 (24.7)	35.5 (27.3)	0.056
LDH	483 (1239)	468 (1003)	480 (1372)	483 (1209)	503 (1343)	0.001
monocyte_count	0.88 (1.33)	0.44 (0.28)	0.72 (0.36)	0.92 (0.40)	1.43 (2.49)	< 0.001 0.979
CRRT						
No	1551 (92.0%)	392 (93.1%)	400 (95.0%)	387 (91.9%)	372 (87.9%)	< 0.001
Yes	135 (8.01%)	29 (6.89%)	21 (4.99%)	34 (8.08%)	51 (12.1%)	0.002
Ventilation						
No	311 (18.4%)	81 (19.2%)	93 (22.1%)	77 (18.3%)	60 (14.2%)	
Yes	1375 (81.6%)	340 (80.8%)	328 (77.9%)	344 (81.7%)	363 (85.8%)	0.029
Sa:						
No	623 (37.0%)	139 (33.0%)	166 (39.4%)	165 (39.2%)	153 (36.2%)	
Yes	1063 (63.0%)	282 (67.0%)	255 (60.6%)	256 (60.8%)	270 (63.8%)	0.177
VP:						
No	636 (37.7%)	160 (38.0%)	161 (38.2%)	175 (41.6%)	140 (33.1%)	
Yes	1050 (62.3%)	261 (62.0%)	260 (61.8%)	246 (58.4%)	283 (66.9%)	0.087
GC						
No	1197 (71.0%)	318 (75.5%)	304 (72.2%)	294 (69.8%)	281 (66.4%)	
Yes	489 (29.0%)	103 (24.5%)	117 (27.8%)	127 (30.2%)	142 (33.6%)	0.028
ABX						
No	20 (1.19%)	7 (1.66%)	6 (1.43%)	4 (0.95%)	3 (0.71%)	
Yes	1666 (98.8%)	414 (98.3%)	415 (98.6%)	417 (99.0%)	420 (99.3%)	0.548
Hosp_Day	22.6 (22.5)	21.2 (23.1)	21.6 (21.1)	24.3 (24.4)	23.5 (21.4)	
ICU_Day	7.27 (8.64)	5.58 (6.93)	6.99 (8.11)	8.36 (10.2)	8.12 (8.76)	
HOSP_Deaths						0.155
No	1493 (88.6%)	389 (92.4%)	383 (91.0%)	371 (88.1%)	350 (82.7%)	< 0.001
Yes	193 (11.4%)	32 (7.60%)	38 (9.03%)	50 (11.9%)	73 (17.3%)	< 0.001
ICU_Deaths						
No	1456 (86.4%)	386 (91.7%)	381 (90.5%)	358 (85.0%)	331 (78.3%)	
Yes	230 (13.6%)	35 (8.31%)	40 (9.50%)	63 (15.0%)	92 (21.7%)	< 0.001

Data are presented as mean ± standard deviation or n (%). AKI, Acute Kidney Injury; APTT, Activated Partial Thromboplastin Time; APACHE II, Acute Physiology and Chronic Health Evaluation II; APS III, Acute Physiology Score III; ALT, Alanine Aminotransferase; ALB, Albumin; AG, Anion Gap; ABX, Antibiotics; AST, Aspartate Aminotransferase; BUN, Blood Urea Nitrogen; CCI, Charlson Comorbidity Index; Cl, Chloride; CKD, Chronic Kidney Disease; COPD, Chronic Obstructive Pulmonary Disease; CRRT, Continuous Renal Replacement Therapy; CRE, Creatinine; DM, Diabetes Mellitus; GC, Glucocorticoid Use; Glu, Glucose; HF, Heart Failure; HR, Heart Rate; HCT, Hematocrit; Hb, Hemoglobin; Hosp, Hospital; HLD, Hyperlipidemia; ICU, Intensive Care Unit; INR, International Normalized Ratio; IHD, Ischemic Heart Disease; Lac, Lactate; LDH, Lactate Dehydrogenase; NBPD, Non-Invasive Blood Pressure—Diastolic; NBPM, Non-Invasive Blood Pressure—Mean; NBPS, Non-Invasive Blood Pressure—Systolic; OASIS, Oxford Acute Severity of Illness Score; Partial Pressure of PCO_2_, Carbon Dioxide; PO_2_, Partial Pressure of Oxygen; SpO_2_, Peripheral Oxygen Saturation; PLT, Platelet; pH, Potential of Hydrogen; K, Potassium; PT, Prothrombin Time; RBC, Red Blood Cell; RDW, Red Cell Distribution Width; RR, Respiratory Rate; SOFA, Sequential Organ Failure Assessment; SAPS II, Simplified Acute Physiology Score II; Na, Sodium; TB, Total Bilirubin; TCa, Total Calcium; TCO_2_, Total Carbon Dioxide; Sa, Vasopressor Administration; VP, Ventilation, Ventricular Pacing; WBC, White Blood Cell.

### Definition of PIV and endpoint

The PIV was calculated using the following formula: [neutrophil count (10^9^/L) × platelet count (10^9^/L) × monocyte count (10^9^/L)]/lymphocyte count (10^9^/L) (19). Given its skewed distribution, the PIV value was log-transformed prior to statistical analysis. For categorical analysis, patients were stratified into four groups (Q1–Q4) based on PIV quartiles. The primary endpoint was 28-day ICU mortality, and the secondary endpoint was 28-day in-hospital all-cause mortality.

### Subgroup analysis

We conducted subgroup analyses to further evaluate the consistency of the association between PIV and the primary outcome across different patient populations. Based on predefined criteria, the analyses included demographic factors [age (≤65 vs. >65 years), sex, and ethnicity] and a range of comorbidity statuses, including hypertension, acute kidney injury (AKI), chronic kidney disease (CKD), diabetes mellitus (DM), hyperlipidemia (HLD), heart failure (HF), ischemic heart disease (IHD), and chronic obstructive pulmonary disease (COPD). Cox proportional hazards models were applied to all subgroups, and the results were visualized using forest plots.

### The correlation between PIV and primary and secondary endpoints

The association between PIV and 28-day all-cause mortality in patients with urosepsis was assessed using multivariable Cox proportional hazards regression models. To control for potential confounders, we constructed three sequentially adjusted models: Model 1 was unadjusted (crude); Model 2 was adjusted for demographic characteristics (age, sex, ethnicity, weight), baseline therapeutic interventions, and pre-existing comorbidities; Model 3 was further adjusted for variables showing significant differences between survivors and non-survivors, including laboratory parameters, vital signs, and disease severity scores at admission. To mitigate the impact of multicollinearity on model stability, the variance inflation factor (VIF) was calculated for all variables in Model 3, and variables with a VIF > 5 were excluded (see Supplementary Table 4). Subsequently, restricted cubic splines and Kaplan-Meier survival curves were employed to further investigate the potential nonlinear relationship between PIV and 28-day all-cause mortality.

### Selection of characteristic variables and modeling and validation of risk prediction

To develop and validate a streamlined risk prediction model for urosepsis patients. The internal MIMIC-IV cohort was randomly split into a training set and an internal testing set at a 7:3 ratio using the “rsample” package in R (random seed = 1). Within the training set, an ensemble machine learning strategy incorporating five complementary methods was applied to screen candidate predictors (laboratory parameters, vital signs, comorbidities, and demographics): Boruta algorithm, LASSO-Cox regression, random forest (RF), gradient boosting decision trees (GBDT), and support vector machine (SVM). Hyperparameters were fixed as follows to avoid data-driven overfitting: Boruta—confidence level 0.01, 100 permutation runs, Bonferroni correction; LASSO-Cox—100 lambda values, lambda.min threshold, 10-fold cross-validation for lambda selection, deviance loss; RF—random seed 1, 100 trees, max depth 3, min samples split 2, min samples leaf 1; GBDT—random seed 1, squared error loss, learning rate 0.1, 100 boosting stages, Friedman mse split criterion, min samples split 2, min samples leaf 1; SVM—random seed 1, regularization parameter C = 1.0, rbf kernel. All feature selection steps were performed exclusively on the training set to prevent information leakage. Only predictors selected by all five methods (consensus variables) were retained for further analysis. A multivariable Cox proportional hazards regression model was then built using these consensus variables on the training set, and the final model was locked (fixed coefficients) before any evaluation on the testing set or external validation cohort. To further assess internal stability, 10-fold cross-validation was performed on the training set using the final five-predictor Cox model, and the cross-validated area under the curve (AUC) was calculated as the average of the 10-folds. Model discrimination was evaluated using ROC curves and AUC in the training set, internal testing set, and external validation cohort, and calibration was assessed using calibration plots.

### Statistical analysis

Continuous variables are presented as mean ± standard deviation (SD) and were compared using the Student’s *t*-test, analysis of variance (ANOVA), Mann–Whitney U test, or Kruskal–Wallis test, as appropriate based on their distribution. Categorical variables are 99expressed as numbers with percentages and were compared using Pearson’s chi-square test or Fisher’s exact test. All statistical analyses were performed using R software (version 4.2.2; RStudio) and the DecisionLinnc package (version 1.0).^1^ A two-sided *p* < 0.05 was considered statistically significant.

## Results

### Baseline characteristics of study participants

This study included 1,686 patients with urosepsis from the MIMIC-IV database. The overall 28-day ICU and in-hospital mortality rates were 13.6% and 11.4%, respectively. Patient groupings based on 28-day ICU and in-hospital mortality status are detailed in Supplementary Tables 1, 2. Baseline characteristics stratified by the PIV quartiles (Q1–Q4, with approximately 421–423 patients per group) are presented in Table 1. The median log-transformed PIV was 2.97, with significant differences observed across quartiles (*P* < 0.001). The mean patient age was 69.6 years, and 997 (59.1%) were female. The prevalence of key comorbidities—acute kidney injury (AKI), chronic kidney disease (CKD), heart failure (HF), and ischemic heart disease (IHD)—increased progressively with higher PIV quartiles (e.g., AKI: 45.8% in Q1 to 58.2% in Q4; all *P* < 0.05). Similarly, most disease severity scores (SOFA, APS III, SAPS II, OASIS, APACHE II, and CCI) showed significant stepwise elevations across PIV groups (all *P* < 0.05). Laboratory profiles revealed that compared to the Q1 group, higher PIV quartiles were associated with lower lymphocyte counts and elevated levels of neutrophils, monocytes, white blood cells, platelets, anion gap, blood glucose, creatinine, and blood urea nitrogen (all *P* < 0.05). Regarding treatments, the use of continuous renal replacement therapy (CRRT) and mechanical ventilation also rose significantly with increasing PIV (CRRT: 6.89% in Q1 to 12.1% in Q4; mechanical ventilation: 80.8% to 85.8%). Consequently, both 28-day ICU mortality and in-hospital mortality increased steadily across PIV quartiles (ICU mortality: 8.31% in Q1 to 21.7% in Q4; in-hospital mortality: 7.60% to 17.3%; both *P* < 0.001).

### Multivariable Cox regression analysis of the association between PIV and 28-day mortality in patients with urosepsis

**FIGURE 1:**
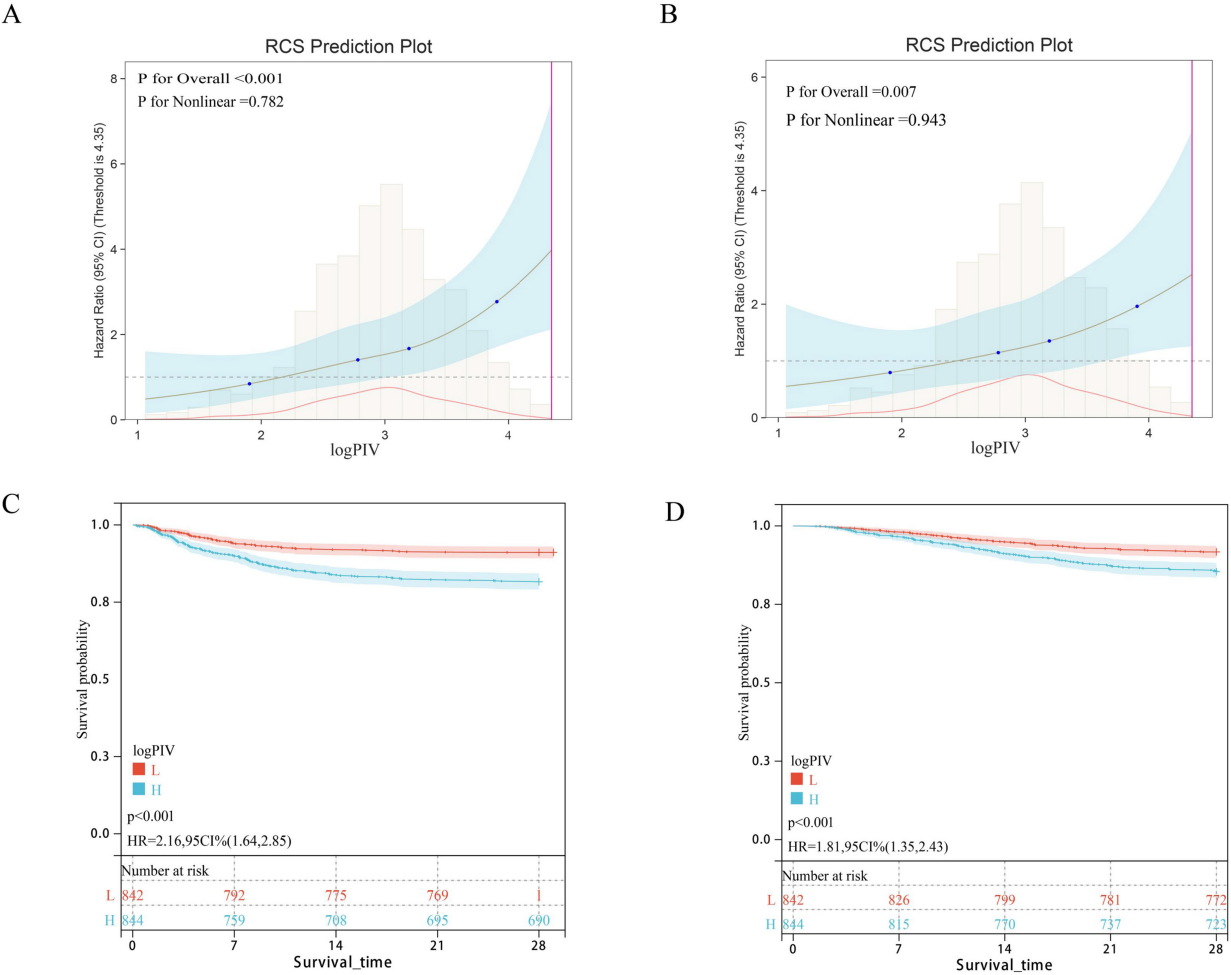
Association between PIV and 28-day mortality in patients with urosepsis. **(A,B)** Restricted cubic spline plots depicting the dose-response relationship of PIV (as a continuous variable) with the risk of **(A)** 28-day ICU mortality and **(B)** 28-day in-hospital mortality. The model was adjusted for all covariates listed in Model 3 of Table 2 (age, sex, race, weight, comorbidities, laboratory parameters, vital signs, disease severity scores, and treatment interventions). The solid line represents the adjusted hazard ratio (HR), and the shaded area indicates the 95% confidence interval (CI). The reference value (HR = 1) is set at the median PIV. The overall association *p*-value (for the nonlinear test) was derived from a likelihood ratio test comparing the model with the linear term only versus the model with restricted cubic splines (3 knots at the 10th, 50th, and 90th percentiles of PIV). **(C,D)** Kaplan–Meier survival curves comparing cumulative survival probability between the high-PIV group (defined as PIV above the median) and the low-PIV group (PIV below the median) for **(C)** 28-day ICU survival and **(D)** 28-day in-hospital survival. The hazard ratio (HR) with 95% CI for the high-PIV group versus the low-PIV group was calculated using a univariate Cox regression model. *P*-values were derived from the log-rank test.

As detailed in Table 2, PIV demonstrated a significant association with both 28-day ICU and in-hospital all-cause mortality in multivariable Cox regression models, irrespective of being analyzed as a continuous or categorical variable. This association persisted across progressively adjusted models (Models 1–3). In the fully adjusted model (Model 3), each unit increase in continuous PIV was associated with a 76.4% higher risk of 28-day ICU mortality (HR = 1.764, 95% CI: 1.340–2.323, *p* < 0.001) and a 63.9% higher risk of 28-day in-hospital mortality (HR = 1.639, 95% CI: 1.214–2.214, *p* = 0.001). When analyzed by quartiles, patients in the highest PIV quartile (Q4) had significantly increased risks of mortality compared to those in the lowest quartile (Q1), with an HR of 1.843 (95% CI: 1.186–2.846, *p* = 0.007) for ICU mortality and an HR of 1.789 (95% CI: 1.110–2.885, *p* = 0.039) for in-hospital mortality. A significant dose-response relationship was confirmed by the trend test (p for trend < 0.05). Restricted cubic spline analysis revealed a significant, monotonically increasing positive association between PIV and 28-day mortality risk (overall *p* < 0.001; Figures 1A,B). Kaplan-Meier survival curves further validated this relationship, showing a significantly lower 28-day cumulative survival rate in the high-PIV group compared to the low-PIV group (Log-rank *p* < 0.001; Figures 1C,D).

**TABLE 2:** The relationship between LAR score and 28 day in-ICU/hospital mortality rate.

Characteristic	Model 1			Model 2			Model 3		
	HR	95% CI	*p*-value	HR	95% CI	*p*-value	HR	95% CI	*p*-value
28-day in-ICU mortality									
Continuous PIV	2.252	1.785, 2.841	< 0.001	1.927	1.533, 2.422	< 0.001	1.764	1.340, 2.323	< 0.001
PIV group									
Q1	Ref	Ref	Ref	Ref	Ref	Ref	Ref	Ref	Ref
Q2	1.153	0.733, 1.815	0.538	1.139	0.722,1.797	0.576	1.086	0.680, 1.733	0.731
Q3	1.862	1.232, 2.815	0.003	1.748	1.152, 2.652	0.009	1.558	1.009, 2.406	0.045
Q4	2.812	1.905, 4.150	< 0.001	2.316	1.560, 3.438	< 0.001	1.843	1.186, 2.846	0.007
28-day in-hospital mortality									
Continuous PIV	1.737	1.355, 2.228	< 0.001	1.604	1.252 2.057	< 0.001	1.639	1.214, 2.214	< 0.001
PIV group									
Q1	Ref	Ref	Ref	Ref	Ref	Ref	Ref	Ref	Ref
Q2	1.098	0.686, 1.758	0.696	1.059	0.659 1.701	0.027	1.167	0.731, 1.911	0.538
Q3	1.328	0.852, 2.070	0.211	1.241	0.793, 1.943	0.345	1.266	0.791, 2.025	0.325
Q4	2.938	1.279, 2.937	0.002	1.735	1.138, 2.647	0.010	1.789	1.110, 2.885	0.039

CI, Confidence Interval; HR, Hazard Ratio. Model 1: unadjusted; Model 2: Adjusted for demographic characteristics (age, gender, race, and weight); baseline comorbidities and treatment interventions. Model 3: Further adjusted for variables that showed significant differences between survivors and non survivors (laboratory tests, vital sign indicators, disease severity score at admission, and treatment interventions).

### Subgroup analysis

**FIGURE 2:**
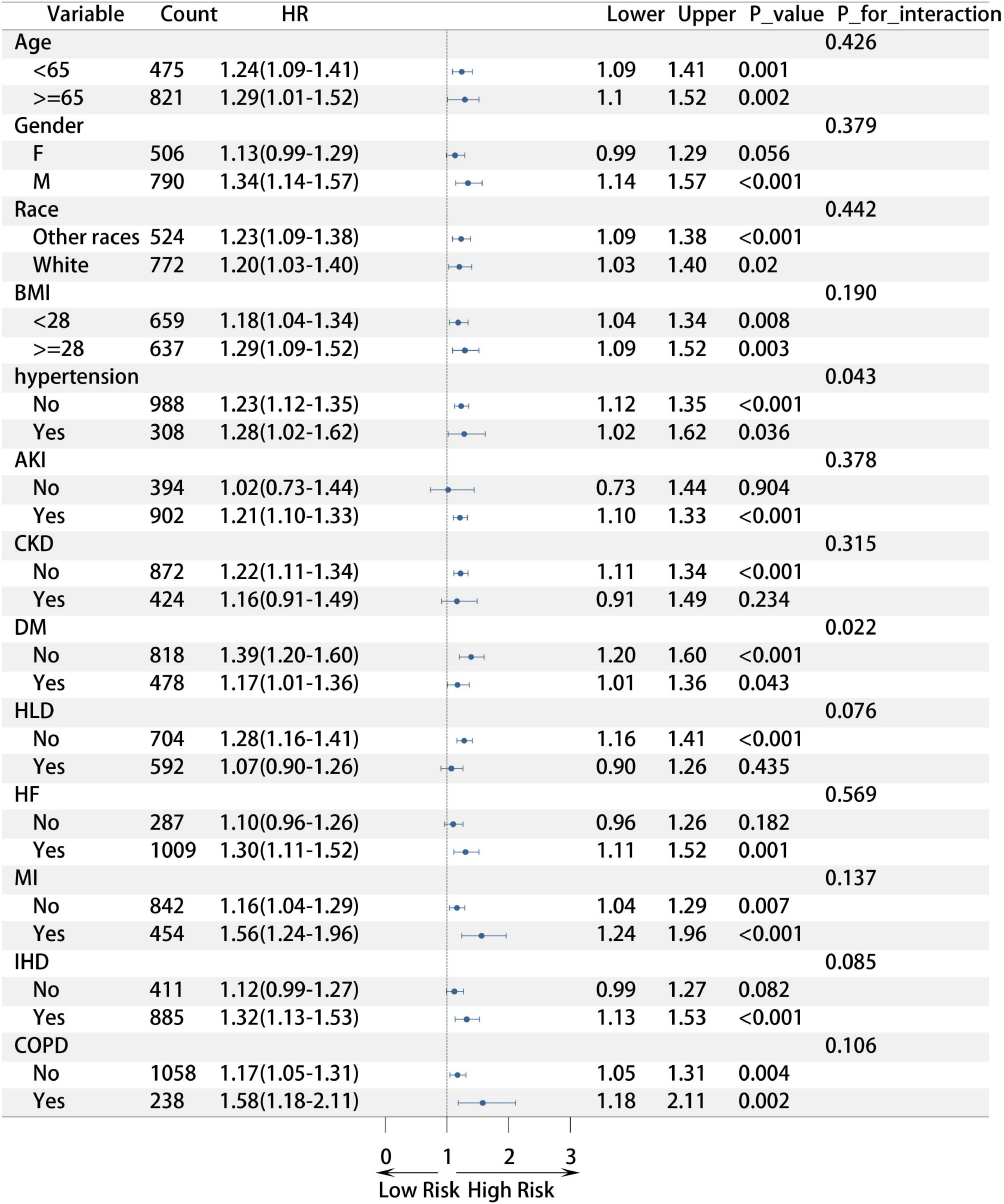
Subgroup analysis of the association between PIV and 28-day ICU mortality. Forest plot of hazard ratios for 28-day ICU mortality associated with a one-unit increase in log-transformed PIV across prespecified subgroups. Subgroup definitions: age ( ≤ 65 vs. > 65 years); sex (female vs. male); race (White vs. other races); comorbidities (hypertension, AKI, CKD, DM, HLD, HF, IHD, COPD) were defined based on ICD codes (MIMIC-IV) or clinical criteria (external cohort). For each subgroup, a separate multivariable Cox regression model was performed adjusted for the same set of covariates as Model 3 in Table 2 (excluding the stratification variable). The square represents the point estimate of the hazard ratio (HR), the horizontal line indicates the 95% confidence interval (CI), and the diamond summarizes the overall effect. The size of the square corresponds to the sample size of the subgroup. Interaction *P*-values were calculated by adding a product term (PIV × subgroup indicator) to the fully adjusted Cox model. *P*-values for the association within each subgroup and *P*-values for interaction are presented.

Subgroup analyses were performed to assess the robustness and heterogeneity of the association between PIV and 28-day ICU mortality across populations with different clinical characteristics (Figure 2). Prespecified subgroups included age, sex, ethnicity, and a range of comorbidities (hypertension, AKI, CKD, diabetes, HLD, HF, IHD, and COPD). The positive association (HR > 1) was consistent in the vast majority of subgroups. Interaction analysis revealed significant effect modification by CKD status (P for interaction = 0.031). Specifically, the predictive effect of PIV was markedly stronger in patients with CKD (HR = 2.92, 95% CI: 1.53–5.57, *P* = 0.001) compared to those without CKD, where the association was not statistically significant (HR = 1.27, 95% CI: 0.92–1.76, *P* = 0.144). Furthermore, PIV remained a significant independent predictor in several other key subgroups: patients aged ≥ 65 years (HR = 1.89, *P* = 0.001), males (HR = 1.80, *P* = 0.014), white people (HR = 2.05, *P* < 0.001), and those with HF (HR = 1.78, *P* = 0.01) or IHD (HR = 1.73, *P* = 0.016). No statistically significant association was observed in subgroups of patients with hypertension (HR = 0.96, *P* = 0.895) or without a history of AKI (HR = 1.23, *P* = 0.502). For all other subgroups (e.g., age < 65 years, females, non-White ethnicities, and patients without diabetes, HLD, or COPD), the interaction *P*-values were greater than 0.05, indicating a consistent positive association between PIV and mortality risk without significant heterogeneity.

### External cohort verification

**FIGURE 3:**
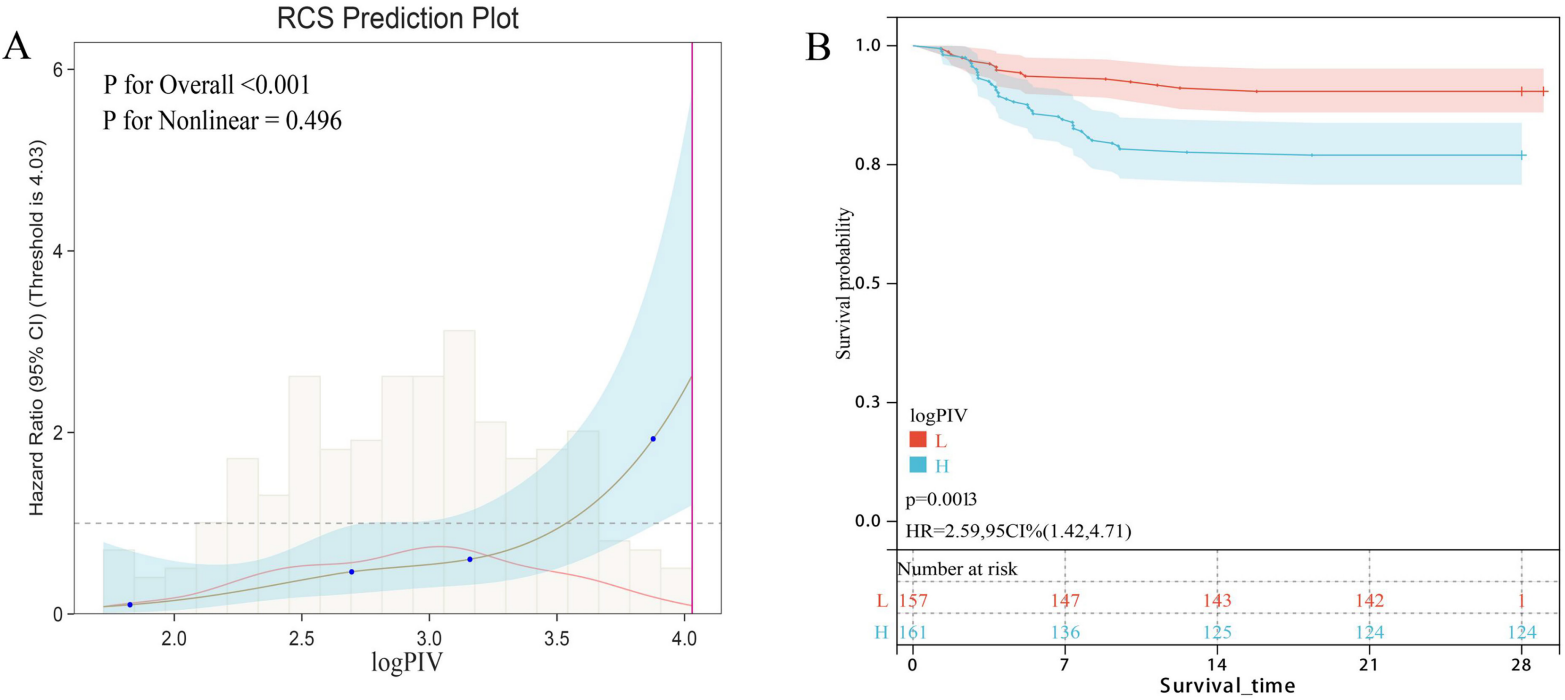
External validation of the association between PIV and 28-day ICU mortality in the independent cohort. **(A)** Restricted cubic spline plot illustrating the dose-response relationship between PIV (as a continuous variable) and the risk of 28-day ICU mortality. The model was adjusted for the same covariates as in the primary analysis (age, sex, comorbidities, laboratory parameters, vital signs, and SOFA score). The solid line represents the adjusted hazard ratio (HR), and the shaded area indicates the 95% confidence interval (CI). The reference value (HR = 1) is set at the median PIV of the external cohort. The overall association *p*-value was derived from a likelihood ratio test using restricted cubic splines with 3 knots (10th, 50th, 90th percentiles). **(B)** Kaplan-Meier survival curves comparing cumulative survival probability between the high-PIV group (PIV above median) and the low-PIV group (PIV below median). The hazard ratio (HR) with 95% CI was calculated using a univariate Cox regression model, and the p-value was derived from the log-rank test.

To validate the stability and applicability of the PIV-based prediction model across different clinical settings, an independent external validation cohort comprising 318 patients with urosepsis was enrolled from Anshun Municipal People’s Hospital. In this cohort, the 28-day ICU mortality rate was 16.35% (Supplementary Table 3). As presented in Table 3, multivariable Cox regression analysis demonstrated that after adjusting for potential confounders, continuous PIV remained significantly associated with an increased risk of 28-day ICU mortality (HR = 1.79, 95% CI: 1.21–2.64, *P* = 0.004). Compared to the lowest quartile (Q1), patients in the highest PIV quartile (Q4) exhibited a substantially elevated mortality risk (HR = 3.12, 95% CI: 1.55–6.27, *P* = 0.001). A significant dose-response relationship was further confirmed by the trend test (P for trend < 0.001). Consistent with the internal training cohort, restricted cubic spline analysis revealed a significant, monotonically increasing positive association between PIV and 28-day mortality risk in the external cohort (overall *P* < 0.001; Figure 3). Kaplan-Meier survival analysis also corroborated these findings, showing a significantly lower 28-day cumulative survival rate among patients with higher PIV levels (Log-rank *P* < 0.001).

**TABLE 3:** The relationship between PIV and external verification cohort 28 day ICU mortality rate.

Characteristic	Model 1			Model 2			Model 3		
	HR	95% CI	*p*-value	HR	95% CI	*p*-value	HR	95% CI	*p*-value
Continuous PIV	3.317	2.037, 5.403	< 0.001	3.259	1.943, 5.469	< 0.001	4.210	2.156, 8.218	< 0.001
PIV group									
Q1	Ref	Ref	Ref	Ref	Ref	Ref	Ref	Ref	Ref
Q2	4.251	1.200, 15.066	0.538	3.994	1.122, 14.219	0.576	2.921	0.721, 11.841	0.113
Q3	4.949	1.422, 17.224	0.003	4.905	1.403, 17.152	0.013	4.485	1.110, 18.128	0.035
Q4	8.689	2.608, 28.947	< 0.001	7.837	2.322, 26.449	0.001	7.383	1.888, 28.869	0.004

CI, confidence interval; HR, hazard ratio. Model 1: unadjusted; Model 2: Adjusted for demographic characteristics (age, gender, race, and weight); baseline comorbidities and treatment interventions. Model 3: Further adjusted for variables that showed significant differences between survivors and non survivors (laboratory tests, vital sign indicators, disease severity score at admission, and treatment interventions).

### Construction and validation of predictive models

**FIGURE 4:**
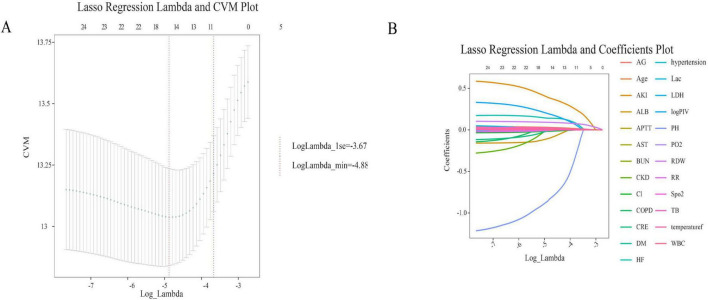
Feature selection using the least absolute shrinkage and selection operator (LASSO) regression. LASSO regression was performed on the training set only (70% of the internal cohort, *n* = 1,180). **(A)** Plot of the cross-validated mean squared error (MSE) against the log(lambda) sequence. Ten-fold cross-validation was used. The left vertical dashed line indicates the lambda value at which the MSE is minimized (lambda.min). The right vertical dashed line indicates the largest lambda value within one standard error of the minimum MSE (lambda.1se), which was selected for constructing a more parsimonious model. **(B)** LASSO coefficient profiles of the candidate variables. Each curve represents the coefficient path of a variable as the penalty term [log(lambda)] increases. The vertical dashed line is drawn at the selected lambda.1se value, where the final set of non-zero coefficients (i.e., selected features) is determined. The outcome variable was 28-day ICU mortality.

**FIGURE 5:**
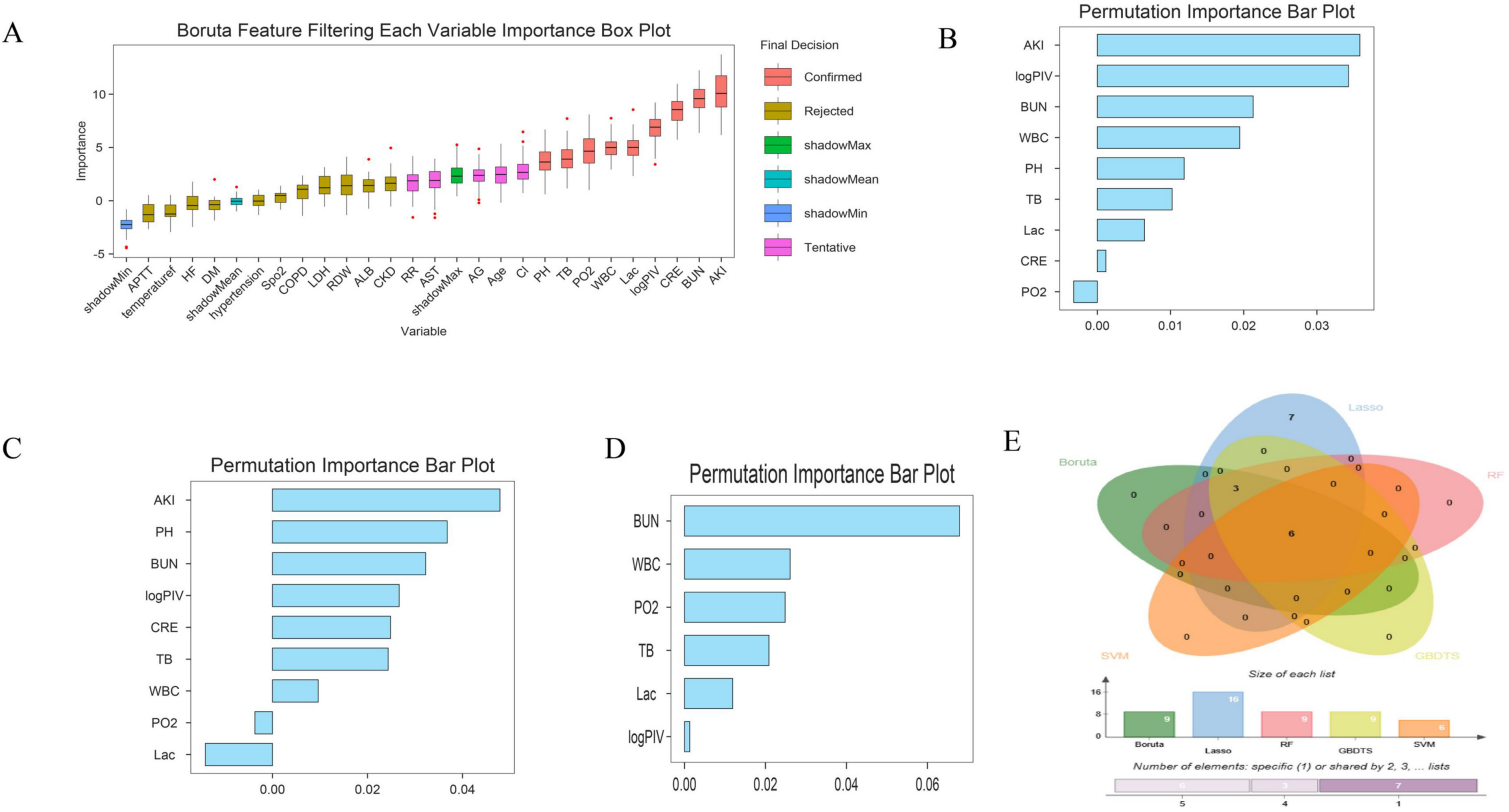
Integrated machine learning feature selection for identifying prognostic variables. All feature selection steps were performed exclusively on the training set (70% of the internal cohort, *n* = 1,180). **(A)** Box plot of variable importance derived from the Boruta algorithm with confidence level = 0.01, 100 permutation runs, and Bonferroni correction. Variables are categorized as “Confirmed” (green), “Tentative” (yellow), or “Rejected” (red) based on their importance compared to random shadow features. **(B–D)** Bar plots of permutation importance from three distinct machine learning models: **(B)** Random Forest (RF) with 100 trees, max depth = 3, min samples split = 2, min samples leaf = 1, random seed = 1; **(C)** Gradient Boosting Decision Trees (GBDT) with squared error loss, learning rate = 0.1, 100 boosting stages, Friedman mse split criterion, min samples split = 2, min samples leaf = 1, random seed = 1; **(D)** Support Vector Machine (SVM) with linear kernel, regularization parameter C = 1.0, random seed = 1. Each bar represents the importance score of a variable for predicting 28-day ICU mortality. **(E)** Venn diagram illustrating the consensus of key variables identified by the five feature selection methods: LASSO regression (Figure 4), Boruta algorithm **(A)**, RF **(B)**, GBDT **(C)**, and SVM **(D)**. The overlapping region highlights the six core prognostic variables (PIV, lactate, PO_2_, total bilirubin, WBC, and BUN) selected by all five methods for final model construction.

**FIGURE 6:**
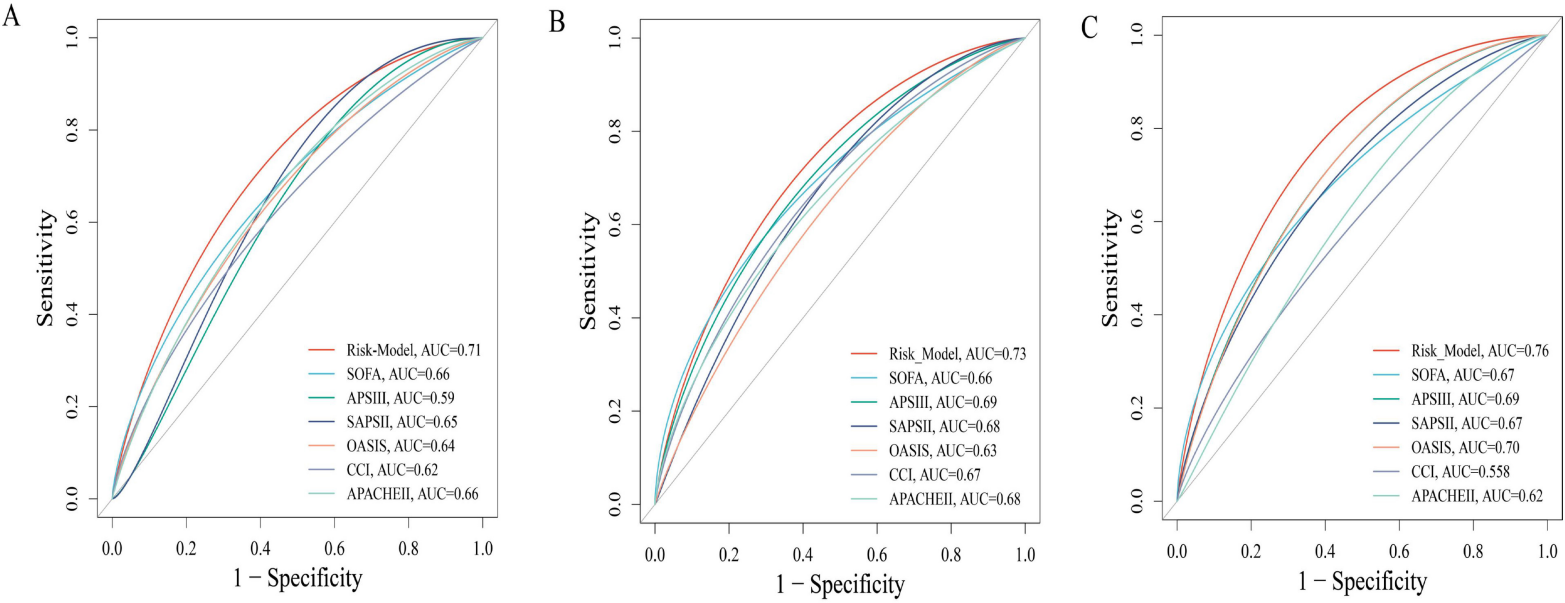
Comparison of model performance using receiver operating characteristic curves across different cohorts. Receiver operating characteristic (ROC) curves evaluating the discriminatory performance for predicting 28-day ICU mortality. ROC curves were generated from the multivariable Cox proportional hazards model using the predicted risk scores. **(A)** Our prognostic model (incorporating PIV, lactate, PO_2_, total bilirubin, and BUN) in the training set [area under the curve (AUC) = 0.71]. **(B)** Our prognostic model in the internal testing set (random 70/30 split of the MIMIC-IV cohort, AUC = 0.73). **(C)** Our prognostic model in the external validation cohort (Anshun Municipal People’s Hospital, *N* = 318, AUC = 0.76). For comparison, the dashed line in each panel represents the performance of a traditional severity score (SOFA in **A,B**; APACHE II in **C**), with its corresponding AUC value displayed. The comparison was performed using DeLong’s test for paired AUCs.

**FIGURE 7:**
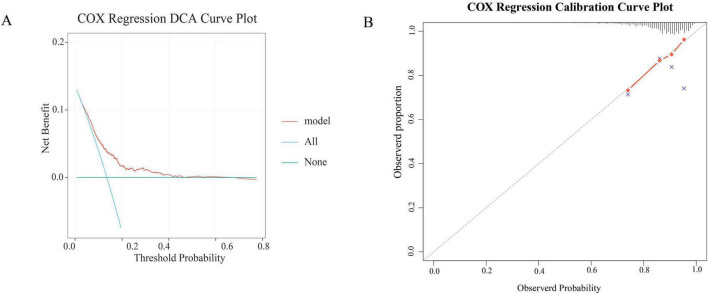
Clinical utility and calibration of the prognostic model. **(A)** Decision curve analysis (DCA) for the prognostic model in the external validation cohort. The y-axis represents the net benefit, and the x-axis represents the threshold probability. The blue curve (“model”) shows the net benefit of using our model (PIV ++ lactate + PO_2_ + total bilirubin + BUN) to guide clinical decisions (e.g., ICU admission, vasopressor initiation) across different probability thresholds. The gray line (“All”) assumes all patients receive the intervention, and the black line (“None”) assumes no patients receive the intervention. Decision curves were calculated using the “dca” package in R, with 28-day ICU mortality as the outcome. The model provides a higher net benefit than both the “treat-all” and “treat-none” strategies across a wide range of threshold probabilities, indicating its potential clinical utility. **(B)** Calibration curve for the prognostic model in the external validation cohort. The y-axis represents the actually observed proportion of events (28-day ICU mortality), and the x-axis represents the predicted probability from the model. Calibration was assessed using the “val.surv” function from the “rms” package, with 200 bootstrap resamples for optimism correction. The blue line (“model”) depicts the relationship between predicted and observed probabilities. The dashed diagonal line represents perfect calibration (ideal line). The close alignment of the model’s calibration curve to the ideal line indicates good agreement between the model’s predictions and the actual outcomes, suggesting reliable predictive accuracy across different risk strata.

The internal cohort was randomly split into training and testing sets at a 7:3 ratio. After excluding variables with significant collinearity (VIF > 5), we evaluated 23 core candidate variables (including laboratory indices, vital signs, demographics, and comorbidities) within the training set using two complementary approaches: LASSO regression (Supplementary Figures 2A,B) and an ensemble of four machine learning algorithms—Random Forest (RF), Boruta algorithm, Gradient Boosting Decision Trees (GBDT), and Support Vector Machine (SVM). The number of key predictors identified by each method was as follows: LASSO: 16 (Figure 4); Boruta: 9 (Figure 5A); RF: 9 (Figure 5B); GBDT: 9 (Figure 5C); and SVM: 6 (Figure 5D). A Venn diagram was used to visualize the consensus among these methods and identify common core prognostic variables (Figure 5E). Subsequent multivariable Cox regression analysis on these common variables confirmed five independent predictors for the final risk prediction model of 28-day ICU mortality: PIV, Lac, PO_2_, TB, and BUN (Table 4). The model demonstrated superior discriminative ability for 28-day ICU mortality compared to traditional severity scores (SOFA, APS III, SAPS II, OASIS, CCI, and APACHE II), achieving area under the receiver operating characteristic curve (AUROC) values of 0.71 (training set; Figure 6A), 0.73 (testing set; Figure 6B), and 0.76 (external validation cohort; Figure 6C). Decision curve analysis (DCA) indicated that, across a wide range of threshold probabilities, the clinical net benefit of using the PIV-based prognostic model substantially exceeded the net benefit of the “treat all” and “treat none” strategies (Figure 7A). This suggests that employing this model for risk stratification and prognosis in clinical practice could yield superior net clinical utility. Furthermore, the calibration curve showed excellent agreement between the predicted and observed probabilities of 28-day ICU mortality across different risk strata, with calibration points closely aligning with the ideal 45-degree line (Figure 7B), indicating reliable predictive accuracy.

**TABLE 4:** Final multivariable Cox proportional hazards model for 28-day mortality.

Variable	coef	exp(coef)	se(coef)	*Z*	*P*-value
logPIV	0.662	1.939	0.140	4.734	<0.001
Lac	0.085	1.089	0.035	2.474	0.013
PO_2_	-0.003	0.997	0.001	-3.050	0.002
TB	0.033	1.033	0.011	3.015	0.003
BUN	0.009	1.009	0.002	3.668	<0.001

Results of the final multivariable Cox proportional hazards regression model for 28-day mortality. BUN, blood urea nitrogen; coef, regression coefficient; exp(coef), hazard ratio; Lac, lactate; logPIV, log-transformed Pan-immune-inflammation Index; PO_2_, partial pressure of oxygen; se(coef), standard error of the coefficient; TB, total bilirubin; *Z*, Z-score.

### Incremental prognostic value of PIV beyond established severity scores

We have now evaluated the incremental prognostic value of PIV beyond six established ICU severity scores (SOFA, APSIII, SAPSII, OASIS, CCI, and APACHE II) using NRI, IDI, and AUROC comparisons (DeLong test). The results are presented in Supplementary Tables 5, 6.

For ICU 28-day mortality, the incremental value was even more pronounced. The AUROC improved from 0.6646 to 0.7139 for SOFA (ΔAUROC = 0.0493, *p* < 0.001), from 0.6664 to 0.7022 for APACHE II (ΔAUROC = 0.0358, *p* = 0.007), and from 0.6358 to 0.6898 for CCI (ΔAUROC = 0.0540, *p* < 0.001). The category-based NRI was 0.2060 (95% CI: 0.1423–0.2683) for SOFA, 0.1366 (95% CI: 0.0736–0.2045) for APACHE II, and 0.1353 (95% CI: 0.0636–0.2057) for CCI. The continuous NRI ranged from 0.3149 to 0.3969, and the IDI from 0.0250 to 0.0361, all with 95% CIs excluding zero.

For hospital 28-day mortality, adding PIV significantly improved discrimination across all severity scores (all ΔAUROC *p* < 0.05). Notably, the AUROC increased from 0.6697 to 0.7084 for SOFA (ΔAUROC = 0.0388, *p* = 0.005), from 0.6571 to 0.6855 for APACHE II (ΔAUROC = 0.0285, *p* = 0.027), and from 0.6333 to 0.6721 for CCI (ΔAUROC = 0.0388, *p* = 0.007). Beyond discrimination, reclassification analyses confirmed clinically meaningful incremental value: the category-based NRI was 0.1873 (95% CI: 0.1325–0.2427) for SOFA, 0.1263 (95% CI: 0.0394–0.2024) for CCI, and 0.1161 (95% CI: 0.0602–0.1774) for APACHE II. The continuous NRI ranged from 0.2815 to 0.3270, and the IDI from 0.0140 to 0.0215, with all 95% CIs excluding zero.

These findings demonstrate that PIV adds significant incremental prognostic information beyond established severity scores, improving not only discrimination but also reclassifying patients into more appropriate risk strata, thereby supporting its clinical utility in existing risk assessment frameworks.

## Discussion

Through a multinational, dual-cohort retrospective analysis, to our knowledge, this is the first study to specifically evaluate PIV in a urosepsis-only cohort for predicting 28-day mortality risk in patients with severe urosepsis. The principal findings are as follows: First, PIV is independently associated with both 28-day ICU and in-hospital mortality, exhibiting a significant and monotonic dose-response relationship. Second, a prognostic model constructed with core variables (PIV, lactate, PO_2_, total bilirubin, and BUN) identified by an ensemble machine learning approach demonstrated stable and superior discriminative performance compared to traditional critical illness scores (e.g., SOFA, APACHE II) in both an internal test set and an independent external validation cohort. Finally, decision curve analysis confirmed that this model offers significant net benefit for clinical decision-making. Collectively, these findings suggest that PIV is not only a promising biomarker but can also serve as a key component of a simple, effective clinical prediction tool to facilitate early risk stratification in patients with urosepsis.

The strong association between PIV and adverse outcomes in urosepsis observed in this study is underpinned by a sound pathophysiological rationale. The PIV formula integrates neutrophil, platelet, monocyte, and lymphocyte counts, thereby concurrently reflecting several key facets of the septic process: elevated neutrophils signify overactivation of innate immunity and systemic inflammatory response; monocytes contribute to the release of inflammatory mediators; platelet activation is closely linked to sepsis-induced endothelial injury and microthrombosis (i.e., immunothrombosis); and lymphocytopenia is a hallmark of sepsis-associated immunosuppression (20–22). Consequently, as a composite metric, PIV offers a more holistic quantification of the severity of the “hyper inflammation immunosuppression-endothelial injury” vicious cycle, although the link to endothelial injury is inferential and PIV does not directly measure it, though it should be noted that PIV does not directly measure endothelial injury, cytokines, or functional immune status; rather, it reflects cellular components that are indirectly linked to these processes. This aligns with our findings that patients with higher PIV exhibited more pronounced neutrophilia, monocytosis, and lymphocytopenia, alongside higher disease severity scores and greater utilization of organ support therapies such as CRRT and mechanical ventilation. Our results are consistent with prior studies in community-acquired pneumonia, intra-abdominal sepsis, and various cancers, thereby extending the applicability of PIV as a biomarker of pan-inflammatory and immune dysregulation (23–25).

Compared to traditional single inflammatory markers (e.g., CRP, PCT) or other hematological composite indices [e.g., neutrophil-tolymphocyte ratio (NLR), platelet-tolymphocyte ratio (PLR)], PIV integrates four cellular components (neutrophils, platelets, monocytes, and lymphocytes) and therefore may provide a broader integrative scope. However, direct comparisons with NLR/PLR using appropriate statistical methods were not performed in the present study, and the suggestion of superiority remains speculative without urosepsis-specific data. The prediction model developed in this study demonstrated stable and consistently superior discriminatory ability (AUC 0.71–0.76) over various traditional critical illness scores. This suggests that combining a core indicator of systemic inflammatory and immune status (PIV) with markers reflecting tissue oxygenation (PO_2_), perfusion and metabolism (lactate), and hepatic/renal function (TB, BUN) may more accurately capture the core pathophysiological disturbances determining prognosis than scores relying solely on physiological parameters. The favorable results from decision curve and calibration analyses further support the model’s clinical applicability, indicating that its risk predictions translate into meaningful net benefit for clinical decision-making and show good agreement with observed outcomes.

Subgroup analyses offer deeper insights for the clinical application of PIV. It is noteworthy that the predictive power of PIV was substantially enhanced in patients with pre-existing chronic kidney disease (CKD). This may be attributed to the baseline chronic inflammatory state and immune dysfunction in CKD patients. When these individuals experience an acute insult such as urosepsis, the dysregulation of their immune-inflammatory system is likely more pronounced, thereby amplifying the derangement captured by PIV. Furthermore, the significant prognostic value of PIV in subgroups such as the elderly, males, White individuals, and those with heart failure or ischemic heart disease suggests that future studies with serial PIV measurements may be particularly warranted in these high-risk populations to determine whether dynamic monitoring provides additional prognostic information beyond baseline values. In contrast, the lack of a significant association in the hypertension subgroup might be related to the anti-inflammatory and immunomodulatory effects of commonly prescribed cardiovascuPIV medications (e.g., statins, ACE inhibitors, or ARBs) in this population, a hypothesis that merits further investigation (26, 27).

This study has several limitations. First, its retrospective observational design cannot entirely rule out residual confounding or establish causality. Second, although a dual-cohort design was employed to enhance generalizability, the internal cohort (MIMIC-IV) originated primarily from a single center in North America and the external cohort from a single center in China. Therefore, the broader applicability of our findings across diverse geographic and ethnic populations requires validation through prospective, multicenter studies with PIV samples. Third, the measurement of PIV and other model variables relied solely on baseline data obtained within 24 h of admission, which does not capture their dynamic trajectories over time. Serial monitoring of these parameters might yield stronger prognostic information. Fourth, although a dual-cohort design was employed to enhance generalizability, both cohorts are derived from single centers—the internal MIMIC-IV cohort from a single tertiary center in North America (Beth Israel Deaconess Medical Center) and the external validation cohort from a single regional hospital in China (Anshun Municipal People’s Hospital). Consequently, external validity to other health systems, practice patterns, and geographic or ethnic populations remains limited.

Fifth, the identification of UTI in the internal MIMIC-IV cohort relied solely on ICD-9/ICD-10 codes, which may lead to diagnostic misclassification. Studies suggest that ICD-based definitions have a sensitivity of approximately 50–70% compared to clinical criteria. However, any such misclassification is likely non-differential with respect to PIV levels, meaning it would bias the results toward the null (i.e., underestimate the true association). Therefore, our observed positive association between PIV and 28-day mortality is likely conservative. Furthermore, the external validation cohort, which used clinical presentation and positive urine culture for diagnosis, showed a similar or even stronger association, reassuring that misclassification in MIMIC-IV did not invalidate our conclusions. Sixth, despite multivariable adjustment for a wide range of potential confounders (demographics, comorbidities, laboratory parameters, and disease severity scores), residual confounding by unmeasured or imperfectly measured variables (e.g., specific uropathogen species, timing and adequacy of antibiotic therapy, genetic polymorphisms influencing immune response) cannot be fully excluded. Additionally, because sepsis is a highly dynamic condition, the observed associations may partially reflect overall disease severity rather than a direct biological effect of PIV. Therefore, our findings should be interpreted as demonstrating association, not causation, and future prospective studies with serial measurements and more detailed clinical phenotyping are needed to further disentangle these relationships. Finally, despite rigorous statistical methods and external validation, the developed model demonstrated moderate predictive accuracy (AUC values), indicating room for improvement. Future research could explore incorporating genomics, proteomics, or more complex temporal data to refine the model.

## Conclusion

This study demonstrates that PIV was independently associated with 28-day mortality after adjustment for major confounders and contributed to a model with moderate discriminative ability (AUC 0.71–0.76). A clinical prediction model integrating PIV with lactate, partial pressure of oxygen, total bilirubin, and blood urea nitrogen demonstrated moderate discriminative ability, acceptable calibration, and net clinical benefit, performing comparably to or slightly better than traditional critical illness scoring systems in the external validation cohort. This model provides clinicians with an easily accessible and cost-effective tool for risk assessment, facilitating the early identification of high-risk patients and enabling timely initiation of more intensive monitoring and personalized interventions. Future prospective, multicenter studies are warranted to validate and refine this model and to explore its potential value in guiding specific treatment strategies.

## Data Availability

The datasets presented in this study can be found in online repositories. The names of the repository/repositories and accession number(s) can be found in the article/Supplementary material.

## Footnotes

1
https://www.statsape.com/
