# Supplementary material for: The relationship between pan immune inflammatory index (PIV) and 28 day mortality in patients with severe urinary sepsis: a retrospective study of a multinational dual cohort study
Source: Front Med (Lausanne). 2026 Jul 10;13:1802352. doi: 10.3389/fmed.2026.1802352 (PMC13395916; doi:10.3389/fmed.2026.1802352)
Supplement: Supplementary file 1 [file Data_Sheet_1.pdf]

TableS1: Baseline characteristics and clinical outcomes of ICU patients by survival status in the internal cohort

Variables	ALL<br>*N=1686*	Survivor<br>*N=1456*	No-survivor<br>*N=230*	P-value
logPIV_group:				<0.001
Q1	421 (25.0%)	386 (26.5%)	35 (15.2%)	
Q2	421 (25.0%)	381 (26.2%)	40 (17.4%)	
Q3	421 (25.0%)	358 (24.6%)	63 (27.4%)	
Q4	423 (25.1%)	331 (22.7%)	92 (40.0%)	
logPIV	2.97 (0.60)	2.93 (0.60)	3.22 (0.59)	<0.001
Age	69.6 (15.6)	69.2 (15.6)	72.1 (15.5)	0.007
Gender:				0.717
F	997 (59.1%)	864 (59.3%)	133 (57.8%)	
M	689 (40.9%)	592 (40.7%)	97 (42.2%)	
Race:				0.420
Other races	703 (41.7%)	601 (41.3%)	102 (44.3%)	
WHITE	983 (58.3%)	855 (58.7%)	128 (55.7%)	
Weight	79.9 (24.4)	79.7 (24.2)	81.1 (25.8)	0.447
hypertension				0.020

Variables	ALL *N=1686*	Survivor *N=1456*	No-survivor *N=230*	P-value
No	1037 (61.5%)	880 (60.4%)	157 (68.3%)	
Yes	649 (38.5%)	576 (39.6%)	73 (31.7%)	
AKI:				<0.001
No	839 (49.8%)	777 (53.4%)	62 (27.0%)	
Yes	847 (50.2%)	679 (46.6%)	168 (73.0%)	
CKD:				0.012
No	1280 (75.9%)	1121 (77.0%)	159 (69.1%)	
Yes	406 (24.1%)	335 (23.0%)	71 (30.9%)	
DM:				0.079
No	1066 (63.2%)	933 (64.1%)	133 (57.8%)	
Yes	620 (36.8%)	523 (35.9%)	97 (42.2%)	
HLD:				0.064
No	957 (56.8%)	813 (55.8%)	144 (62.6%)	
Yes	729 (43.2%)	643 (44.2%)	86 (37.4%)	
HF:				<0.001
No	1139 (67.6%)	1018 (69.9%)	121 (52.6%)	

Variables	ALL *N=1686*	Survivor *N=1456*	No-survivor *N=230*	P-value
Yes	547 (32.4%)	438 (30.1%)	109 (47.4%)	
IHD:				0.078
No	1045 (62.0%)	915 (62.8%)	130 (56.5%)	
Yes	641 (38.0%)	541 (37.2%)	100 (43.5%)	
COPD:				0.014
No	1442 (85.5%)	1258 (86.4%)	184 (80.0%)	
Yes	244 (14.5%)	198 (13.6%)	46 (20.0%)	
SOFA	6.13 (3.44)	5.83 (3.22)	8.07 (4.13)	<0.001
APSIII	50.0 (21.3)	48.0 (20.2)	63.8 (23.1)	<0.001
SAPSII	41.6 (12.9)	41.9 (13.2)	39.8 (11.0)	0.009
OASIS	33.6 (8.25)	33.0 (8.08)	37.2 (8.42)	<0.001
CCI	5.75 (2.85)	5.56 (2.79)	6.97 (2.95)	<0.001
APACHEII	19.7 (7.21)	19.1 (7.01)	23.3 (7.40)	<0.001
HR	89.9 (20.4)	89.8 (20.2)	90.9 (21.8)	0.462
NBPS	122 (24.9)	122 (24.9)	120 (24.8)	0.175
NBPD	70.0 (19.1)	70.0 (18.8)	70.5 (21.4)	0.718

Variables	ALL<br>*N=1686*	Survivor<br>*N=1456*	No-survivor<br>*N=230*	P-value
NBPM	83.8 (19.2)	83.8 (19.1)	83.7 (20.5)	0.978
RR	19.9 (6.29)	19.8 (6.29)	20.6 (6.23)	0.055
Spo2	96.9 (3.80)	97.0 (3.72)	96.2 (4.21)	0.011
temperaturef	98.0 (5.01)	98.0 (4.85)	97.6 (5.93)	0.328
lymphocyte_count	1.40 (1.34)	1.41 (1.10)	1.36 (2.36)	0.771
HCT	31.8 (6.86)	31.9 (6.78)	31.1 (7.31)	0.171
Hb	10.2 (2.27)	10.3 (2.26)	9.98 (2.36)	0.059
PLT	197 (99.5)	197 (97.5)	193 (112)	0.577
RDW	15.3 (2.66)	15.1 (2.51)	16.7 (3.17)	<0.001
RBC	3.47 (0.81)	3.48 (0.79)	3.37 (0.88)	0.067
WBC	13.4 (8.19)	13.0 (7.20)	16.0 (12.5)	<0.001
neutrophil_count	10.7 (7.28)	10.2 (6.65)	13.4 (10.0)	<0.001
ALB	3.02 (0.61)	3.05 (0.60)	2.87 (0.69)	<0.001
AG	14.9 (4.50)	14.7 (4.39)	16.0 (5.01)	<0.001
TCa	8.40 (0.84)	8.41 (0.85)	8.36 (0.79)	0.415
Cl	103 (7.48)	104 (7.26)	101 (8.47)	<0.001

Variables	ALL<br>*N=1686*	Survivor<br>*N=1456*	No-survivor<br>*N=230*	P-value
Glu	152 (79.9)	151 (81.5)	154 (68.8)	0.577
K	4.22 (0.76)	4.21 (0.75)	4.27 (0.82)	0.288
Na	138 (6.40)	139 (6.32)	138 (6.89)	0.279
TCO2	24.4 (5.69)	24.4 (5.48)	23.9 (6.87)	0.253
Lac	2.23 (1.76)	2.17 (1.74)	2.63 (1.87)	0.001
PCO2	41.7 (11.0)	41.5 (10.8)	42.9 (11.8)	0.084
PH	7.37 (0.09)	7.37 (0.09)	7.34 (0.10)	<0.001
PO2	127 (106)	132 (109)	91.7 (70.0)	<0.001
INR	1.50 (0.81)	1.45 (0.71)	1.83 (1.23)	<0.001
PT	16.4 (9.17)	15.8 (8.15)	20.0 (13.5)	<0.001
APTT	37.8 (23.6)	37.2 (23.5)	41.2 (23.6)	0.018
ALT	116 (614)	119 (639)	95.5 (418)	0.458
AST	201 (1090)	208 (1146)	162 (634)	0.372
TB	1.79 (4.68)	1.51 (3.72)	3.54 (8.33)	<0.001
CRE	1.49 (1.38)	1.44 (1.38)	1.80 (1.38)	<0.001
BUN	30.0 (25.0)	28.2 (23.6)	41.1 (30.2)	<0.001

Variables	ALL<br>*N=1686*	Survivor<br>*N=1456*	No-survivor<br>*N=230*	P-value
LDH	483 (1239)	474 (1207)	546 (1427)	0.467
monocyte_count	0.88 (1.33)	0.81 (0.52)	1.30 (3.33)	0.028
CRRT:				<0.001
No	1551 (92.0%)	1365 (93.8%)	186 (80.9%)	
Yes	135 (8.01%)	91 (6.25%)	44 (19.1%)	
Ventilation:				0.029
No	311 (18.4%)	281 (19.3%)	30 (13.0%)	
Yes	1375 (81.6%)	1175 (80.7%)	200 (87.0%)	
Sa:				<0.001
No	623 (37.0%)	577 (39.6%)	46 (20.0%)	
Yes	1063 (63.0%)	879 (60.4%)	184 (80.0%)	
VP:				<0.001
No	636 (37.7%)	595 (40.9%)	41 (17.8%)	
Yes	1050 (62.3%)	861 (59.1%)	189 (82.2%)	
GC:				<0.001
No	1197 (71.0%)	1065 (73.1%)	132 (57.4%)	

Variables	ALL<br>*N=1686*	Survivor<br>*N=1456*	No-survivor<br>*N=230*	P-value
Yes	489 (29.0%)	391 (26.9%)	98 (42.6%)	
ABX:				0.097
No	20 (1.19%)	20 (1.37%)	0 (0.00%)	
Yes	1666 (98.8%)	1436 (98.6%)	230 (100%)	

Note: Data are presented as mean ± standard deviation or n (%). Abbreviations: Acute Kidney Injury (AKI), Activated Partial Thromboplastin Time (APTT), Acute Physiology and Chronic Health Evaluation II (APACHE II), Acute Physiology Score III (APS III), Alanine Aminotransferase (ALT), Albumin (ALB), Anion Gap (AG), Antibiotics (ABX), Aspartate Aminotransferase (AST), Blood Urea Nitrogen (BUN), Charlson Comorbidity Index (CCI), Chloride (Cl), Chronic Kidney Disease (CKD), Chronic Obstructive Pulmonary Disease (COPD), Continuous Renal Replacement Therapy (CRRT), Creatinine (CRE), Diabetes Mellitus (DM), Glucocorticoid Use (GC), Glucose (Glu), Heart Failure (HF), Heart Rate (HR), Hematocrit (HCT), Hemoglobin (Hb), Hospital (Hosp), Hyperlipidemia (HLD), Intensive Care Unit (ICU), International Normalized Ratio (INR), Ischemic Heart Disease (IHD), Lactate (Lac), Lactate Dehydrogenase (LDH), Non-Invasive Blood Pressure—Diastolic (NBPD), Non-Invasive Blood Pressure—Mean (NBPM), Non-Invasive Blood Pressure—Systolic (NBPS), Oxford Acute Severity of Illness Score (OASIS), Partial Pressure of Carbon Dioxide (PCO_2), Partial Pressure of Oxygen (PO_2), Peripheral Oxygen Saturation (SpO_2), Platelet (PLT), Potential of Hydrogen (pH), Potassium (K), Prothrombin Time (PT), Red Blood Cell (RBC), Red Cell Distribution Width (RDW), Respiratory Rate (RR), Sequential Organ Failure Assessment (SOFA), Simplified Acute Physiology Score II (SAPS II), Sodium (Na), Total Bilirubin (TB), Total Calcium (TCa), Total Carbon Dioxide (TCO_2), Vasopressor Administration (Sa), Ventilation, Ventricular Pacing (VP), White Blood Cell (WBC).

TableS2: Baseline characteristics and clinical outcomes of in-28day hospital patients by survival status in the internal cohor

Variables	ALL<br>*N=1686*	Survivor<br>*N=1493*	No-survivor<br>*N=193*	P-value
logPIV_group:				<0.001
Q1	421 (25.0%)	389 (26.1%)	32 (16.6%)	
Q2	421 (25.0%)	383 (25.7%)	38 (19.7%)	
Q3	421 (25.0%)	371 (24.8%)	50 (25.9%)	
Q4	423 (25.1%)	350 (23.4%)	73 (37.8%)	
logPIV	2.97 (0.60)	2.94 (0.60)	3.18 (0.59)	<0.001
Age	69.6 (15.6)	69.2 (15.6)	72.2 (15.8)	0.015
Gender:				0.471
F	997 (59.1%)	888 (59.5%)	109 (56.5%)	
M	689 (40.9%)	605 (40.5%)	84 (43.5%)	
Race:				0.436
Other races	703 (41.7%)	617 (41.3%)	86 (44.6%)	
WHITE	983 (58.3%)	876 (58.7%)	107 (55.4%)	

Variables	ALL<br>*N=1686*	Survivor<br>*N=1493*	No-survivor<br>*N=193*	P-value
Weight	79.9 (24.4)	79.7 (24.1)	81.6 (26.5)	0.338
hypertension				0.013
No	1037 (61.5%)	903 (60.5%)	134 (69.4%)	
Yes	649 (38.5%)	590 (39.5%)	59 (30.56%)	
AKI:				<0.001
No	839 (49.8%)	784 (52.5%)	55 (28.5%)	
Yes	847 (50.2%)	709 (47.5%)	138 (71.5%)	
CKD:				0.020
No	1280 (75.9%)	1147 (76.8%)	133 (68.9%)	
Yes	406 (24.1%)	346 (23.2%)	60 (31.1%)	
DM:				0.047
No	1066 (63.2%)	957 (64.1%)	109 (56.5%)	
Yes	620 (36.8%)	536 (35.9%)	84 (43.5%)	
HLD:				0.124
No	957 (56.8%)	837 (56.1%)	120 (62.2%)	
Yes	729 (43.2%)	656 (43.9%)	73 (37.8%)	

Variables	ALL<br>*N=1686*	Survivor<br>*N=1493*	No-survivor<br>*N=193*	P-value
HF:				<0.001
No	1139 (67.6%)	1040 (69.7%)	99 (51.3%)	
Yes	547 (32.4%)	453 (30.3%)	94 (48.7%)	
IHD:				0.080
No	1045 (62.0%)	937 (62.8%)	108 (56.0%)	
Yes	641 (38.0%)	556 (37.2%)	85 (44.0%)	
COPD:				0.022
No	1442 (85.5%)	1288 (86.3%)	154 (79.8%)	
Yes	244 (14.5%)	205 (13.7%)	39 (20.2%)	
SOFA	6.13 (3.44)	5.87 (3.25)	8.18 (4.15)	<0.001
APSIII	50.0 (21.3)	48.3 (20.4)	63.4 (23.2)	<0.001
SAPSII	41.6 (12.9)	41.8 (13.1)	39.9 (11.1)	0.030
OASIS	33.6 (8.25)	33.1 (8.14)	37.4 (8.17)	<0.001
CCI	5.75 (2.85)	5.60 (2.80)	6.96 (2.94)	<0.001
APACHEII	19.7 (7.21)	19.2 (7.07)	23.2 (7.30)	<0.001
HR	89.9 (20.4)	89.8 (20.4)	90.7 (20.7)	0.585

Variables	ALL<br>*N=1686*	Survivor<br>*N=1493*	No-survivor<br>*N=193*	P-value
NBPS	122 (24.9)	122 (25.0)	119 (23.8)	0.069
NBPD	70.0 (19.1)	70.1 (19.0)	69.5 (20.4)	0.707
NBPM	83.8 (19.2)	83.9 (19.2)	82.8 (19.4)	0.444
RR	19.9 (6.29)	19.8 (6.28)	20.5 (6.36)	0.140
Spo2	96.9 (3.80)	96.9 (3.76)	96.4 (4.09)	0.088
temperaturef	98.0 (5.01)	98.0 (4.79)	97.6 (6.46)	0.310
lymphocyte_count	1.40 (1.34)	1.40 (1.09)	1.41 (2.56)	0.961
HCT	31.8 (6.86)	31.8 (6.79)	31.6 (7.37)	0.690
Hb	10.2 (2.27)	10.3 (2.26)	10.1 (2.36)	0.435
PLT	197 (99.5)	197 (98.2)	190 (109)	0.408
RDW	15.3 (2.66)	15.1 (2.55)	16.6 (3.12)	<0.001
RBC	3.47 (0.81)	3.47 (0.79)	3.42 (0.90)	0.411
WBC	13.4 (8.19)	13.1 (7.61)	15.3 (11.6)	0.012
neutrophil_count	10.7 (7.28)	10.4 (6.76)	13.1 (10.2)	<0.001
ALB	3.02 (0.61)	3.04 (0.60)	2.89 (0.68)	0.004
AG	14.9 (4.50)	14.7 (4.50)	15.8 (4.42)	0.001

Variables	ALL *N=1686*	Survivor *N=1493*	No-survivor *N=193*	P-value
TCa	8.40 (0.84)	8.41 (0.85)	8.37 (0.74)	0.535
Cl	103 (7.48)	104 (7.29)	101 (8.58)	<0.001
Glu	152 (79.9)	151 (81.5)	155 (66.2)	0.455
K	4.22 (0.76)	4.22 (0.75)	4.25 (0.82)	0.625
Na	138 (6.40)	139 (6.29)	138 (7.19)	0.353
TCO2	24.4 (5.69)	24.4 (5.52)	23.9 (6.90)	0.298
Lac	2.23 (1.76)	2.18 (1.76)	2.59 (1.74)	0.002
PCO2	41.7 (11.0)	41.5 (10.8)	42.8 (12.2)	0.154
PH	7.37 (0.09)	7.37 (0.09)	7.34 (0.10)	<0.001
PO2	127 (106)	131 (108)	92.3 (70.6)	<0.001
INR	1.50 (0.81)	1.45 (0.71)	1.85 (1.29)	<0.001
PT	16.4 (9.17)	15.9 (8.18)	20.2 (14.2)	<0.001
APTT	37.8 (23.6)	37.3 (23.6)	41.5 (22.9)	0.019
ALT	116 (614)	119 (633)	92.0 (439)	0.443
AST	201 (1090)	209 (1137)	144 (620)	0.227
TB	1.79 (4.68)	1.56 (3.92)	3.61 (8.29)	0.001

Variables	ALL *N=1686*	Survivor *N=1493*	No-survivor *N=193*	P-value
CRE	1.49 (1.38)	1.45 (1.38)	1.78 (1.40)	0.002
BUN	30.0 (25.0)	28.6 (24.0)	40.7 (29.6)	<0.001
LDH	483 (1239)	477 (1202)	531 (1500)	0.634
monocyte_count	0.88 (1.33)	0.85 (1.36)	1.08 (1.07)	0.007
CRRT:				<0.001
No	1551 (92.0%)	1393 (93.3%)	158 (81.9%)	
Yes	135 (8.01%)	100 (6.70%)	35 (18.1%)	
Ventilation:				0.029
No	311 (18.4%)	287 (19.2%)	24 (12.4%)	
Yes	1375 (81.6%)	1206 (80.8%)	169 (87.6%)	
Sa:				<0.001
No	623 (37.0%)	583 (39.0%)	40 (20.7%)	
Yes	1063 (63.0%)	910 (61.0%)	153 (79.3%)	
VP:				<0.001
No	636 (37.7%)	601 (40.3%)	35 (18.1%)	
Yes	1050 (62.3%)	892 (59.7%)	158 (81.9%)	

Variables	ALL *N=1686*	Survivor *N=1493*	No-survivor *N=193*	P-value
GC:				<0.001
No	1197 (71.0%)	1085 (72.7%)	112 (58.0%)	
Yes	489 (29.0%)	408 (27.3%)	81 (42.0%)	
ABX:				0.156
No	20 (1.19%)	20 (1.34%)	0 (0.00%)	
Yes	1666 (98.8%)	1473 (98.7%)	193 (100%)	

Note: Data are presented as mean ± standard deviation or n (%). Abbreviations: Acute Kidney Injury (AKI), Activated Partial Thromboplastin Time (APTT), Acute Physiology and Chronic Health Evaluation II (APACHE II), Acute Physiology Score III (APS III), Alanine Aminotransferase (ALT), Albumin (ALB), Anion Gap (AG), Antibiotics (ABX), Aspartate Aminotransferase (AST), Blood Urea Nitrogen (BUN), Charlson Comorbidity Index (CCI), Chloride (Cl), Chronic Kidney Disease (CKD), Chronic Obstructive Pulmonary Disease (COPD), Continuous Renal Replacement Therapy (CRRT), Creatinine (CRE), Diabetes Mellitus (DM), Glucocorticoid Use (GC), Glucose (Glu), Heart Failure (HF), Heart Rate (HR), Hematocrit (HCT), Hemoglobin (Hb), Hospital (Hosp), Hyperlipidemia (HLD), Intensive Care Unit (ICU), International Normalized Ratio (INR), Ischemic Heart Disease (IHD), Lactate (Lac), Lactate Dehydrogenase (LDH), Non-Invasive Blood Pressure—Diastolic (NBPD), Non-Invasive Blood Pressure—Mean (NBPM), Non-Invasive Blood Pressure—Systolic (NBPS), Oxford Acute Severity of Illness Score (OASIS), Partial Pressure of Carbon Dioxide (PCO_2), Partial Pressure of Oxygen (PO_2), Peripheral Oxygen Saturation (SpO_2), Platelet (PLT), Potential of Hydrogen (pH), Potassium (K), Prothrombin Time (PT), Red Blood Cell (RBC), Red Cell Distribution Width (RDW), Respiratory Rate (RR), Sequential Organ Failure Assessment (SOFA), Simplified Acute Physiology Score II (SAPS II), Sodium (Na), Total Bilirubin (TB), Total Calcium (TCa), Total Carbon Dioxide (TCO_2), Vasopressor Administration (Sa), Ventilation, Ventricular Pacing (VP), White Blood Cell (WBC).

Table S3: Summary descriptives table by groups of external verification cohort Dead

Variables	ALL *N=318*	Survivor *N=266*	No-survivor *N=52*	P-value
logPIV	2.92 (0.62)	2.85 (0.61)	3.29 (0.57)	<0.001
logPIV_group:				<0.001
Q1	79 (24.8%)	76 (28.6%)	3 (5.77%)	
Q2	79 (24.8%)	67 (25.2%)	12 (23.1%)	
Q3	79 (24.8%)	65 (24.4%)	14 (26.9%)	
Q4	81 (25.5%)	58 (21.8%)	23 (44.2%)	
Age	69.4 (15.6)	69.0 (15.5)	71.7 (16.2)	0.266
Gender:				1.000
F	196 (61.6%)	164 (61.7%)	32 (61.5%)	
M	122 (38.4%)	102 (38.3%)	20 (38.5%)	
BMI	29.4 (7.89)	29.6 (8.08)	28.6 (6.84)	0.379
hypertension:				0.710
No	210 (66.0%)	174 (65.4%)	36 (69.2%)	
Yes	108 (34.0%)	92 (34.6%)	16 (30.8%)	
AKI:				0.012

Variables	ALL *N=318*	Survivor *N=266*	No-survivor *N=52*	P-value
No	164 (51.6%)	146 (54.9%)	18 (34.6%)	
Yes	154 (48.4%)	120 (45.1%)	34 (65.4%)	
CVA:				0.777
No	295 (92.8%)	247 (92.9%)	48 (92.3%)	
Yes	23 (7.23%)	19 (7.14%)	4 (7.69%)	
CKD:				0.748
No	241 (75.8%)	203 (76.3%)	38 (73.1%)	
Yes	77 (24.2%)	63 (23.7%)	14 (26.9%)	
DM:				0.293
No	189 (59.4%)	162 (60.9%)	27 (51.9%)	
Yes	129 (40.6%)	104 (39.1%)	25 (48.1%)	
HLD:				0.452
No	190 (59.7%)	156 (58.6%)	34 (65.4%)	
Yes	128 (40.3%)	110 (41.4%)	18 (34.6%)	
HF:				0.031
No	215 (67.6%)	187 (70.3%)	28 (53.8%)	

Variables	ALL *N=318*	Survivor *N=266*	No-survivor *N=52*	P-value
Yes	103 (32.4%)	79 (29.7%)	24 (46.2%)	
MI:				1.000
No	269 (84.6%)	225 (84.6%)	44 (84.6%)	
Yes	49 (15.4%)	41 (15.4%)	8 (15.4%)	
IHD:				0.593
No	203 (63.8%)	172 (64.7%)	31 (59.6%)	
Yes	115 (36.2%)	94 (35.3%)	21 (40.4%)	
COPD:				0.394
No	272 (85.5%)	230 (86.5%)	42 (80.8%)	
Yes	46 (14.5%)	36 (13.5%)	10 (19.2%)	
SOFA	6.11 (3.46)	5.68 (3.06)	8.27 (4.46)	<0.001
APSIII	48.9 (20.8)	46.5 (19.5)	61.1 (22.8)	<0.001
SAPSII	39.4 (13.0)	37.9 (12.3)	46.8 (14.1)	<0.001
OASIS	33.1 (8.27)	32.1 (7.99)	38.1 (7.98)	<0.001
CCI	5.75 (2.93)	5.61 (2.85)	6.44 (3.27)	0.091
APACHEII	19.2 (6.96)	18.8 (6.96)	21.5 (6.58)	0.008

**Variables**	**ALL** *N=318*	**Survivor** *N=266*	**No-survivor** *N=52*	**P-value**
HR	90.5 (20.2)	90.0 (18.8)	93.0 (26.5)	0.433
NBPS	120 (23.8)	121 (23.5)	115 (24.7)	0.122
NBPD	70.1 (18.8)	70.4 (18.0)	69.0 (22.8)	0.689
NBPM	83.8 (18.8)	84.3 (18.2)	81.3 (21.4)	0.353
RR	20.5 (6.80)	20.3 (6.73)	21.7 (7.11)	0.197
Spo2	96.9 (3.84)	97.1 (3.66)	95.6 (4.46)	0.021
lymphocyte_count	1.34 (0.93)	1.40 (0.95)	1.01 (0.72)	0.001
HCT	31.6 (6.90)	31.4 (6.85)	32.5 (7.13)	0.303
Hb	10.2 (2.29)	10.1 (2.29)	10.5 (2.29)	0.303
PLT	186 (95.9)	181 (88.0)	210 (127)	0.130
RDW	15.5 (2.71)	15.2 (2.50)	16.8 (3.33)	0.002
RBC	3.46 (0.82)	3.44 (0.82)	3.57 (0.81)	0.284
WBC	13.0 (7.37)	12.7 (7.17)	15.0 (8.13)	0.062
neutrophil_count	10.3 (6.61)	9.67 (6.05)	13.6 (8.29)	0.002
ALB	3.01 (0.60)	3.04 (0.60)	2.85 (0.62)	0.042
AG	15.0 (4.64)	14.8 (4.61)	16.1 (4.66)	0.054

Variables	ALL *N=318*	Survivor *N=266*	No-survivor *N=52*	P-value
TCa	8.40 (0.79)	8.39 (0.81)	8.45 (0.71)	0.579
Cl	104 (7.22)	104 (7.32)	102 (6.25)	0.006
Glu	141 (65.4)	138 (59.2)	159 (89.3)	0.111
K	4.22 (0.84)	4.23 (0.85)	4.13 (0.74)	0.388
Na	139 (6.09)	139 (6.26)	138 (5.10)	0.281
TCO2	24.0 (5.47)	24.0 (5.46)	24.2 (5.56)	0.775
Lac	2.17 (1.73)	2.08 (1.75)	2.68 (1.56)	0.014
PCO2	40.8 (10.4)	40.1 (10.2)	44.0 (10.9)	0.021
PH	7.37 (0.09)	7.37 (0.08)	7.34 (0.11)	0.037
PO2	123 (107)	132 (112)	79.5 (60.2)	<0.001
INR	1.56 (0.82)	1.48 (0.72)	1.95 (1.14)	0.005
PT	17.0 (9.10)	16.1 (7.99)	21.4 (12.6)	0.005
APTT	39.2 (24.5)	37.3 (22.0)	49.0 (33.0)	0.016
ALT	133 (566)	124 (504)	183 (816)	0.619
AST	234 (1051)	225 (1036)	279 (1136)	0.751
TB	2.13 (5.00)	1.83 (4.21)	3.67 (7.78)	0.104

Variables	ALL<br>*N=318*	Survivor<br>*N=266*	No-survivor<br>*N=52*	P-value
CRE	1.41 (1.36)	1.42 (1.39)	1.38 (1.20)	0.830
BUN	28.5 (23.9)	28.1 (23.9)	30.8 (24.0)	0.452
LDH	484 (1043)	492 (1122)	446 (461)	0.630
monocyte_count	0.82 (0.52)	0.79 (0.51)	0.95 (0.57)	0.064
Ventilation:				0.131
No	57 (17.9%)	52 (19.5%)	5 (9.62%)	
Yes	261 (82.1%)	214 (80.5%)	47 (90.4%)	
Sa:				0.073
No	124 (39.0%)	110 (41.4%)	14 (26.9%)	
Yes	194 (61.0%)	156 (58.6%)	38 (73.1%)	
VP:				0.003
No	115 (36.2%)	106 (39.8%)	9 (17.3%)	
Yes	203 (63.8%)	160 (60.2%)	43 (82.7%)	
GC:				0.175
No	235 (73.9%)	201 (75.6%)	34 (65.4%)	
Yes	83 (26.1%)	65 (24.4%)	18 (34.6%)	

Variables	ALL *N=318*	Survivor *N=266*	No-survivor *N=52*	P-value
ABX:				1.000
No	3 (0.94%)	3 (1.13%)	0 (0.00%)	
Yes	315 (99.1%)	263 (98.9%)	52 (100%)	
Survival_time	24.4 (8.41)	28.0 (0.06)	5.68 (3.76)	<0.001

Data are presented as mean ± standard deviation or n (%). Abbreviations:Body Mass Index (BMI), Acute Kidney Injury (AKI), Chronic Kidney Disease (CKD), Diabetes Mellitus (DM), Hyperlipidemia (HLD), Heart Failure (HF), Ischemic Heart Disease (IHD), Chronic Obstructive Pulmonary Disease (COPD), Heart Rate (HR), Non-Invasive Blood Pressure—Systolic, Diastolic, Mean (NBPS, NBPD, NBPM), Respiratory Rate (RR), Peripheral Oxygen Saturation (SpO_2), Sequential Organ Failure Assessment (SOFA), Acute Physiology Score III (APS III), Simplified Acute Physiology Score II (SAPS II), Oxford Acute Severity of Illness Score (OASIS), Charlson Comorbidity Index (CCI), Acute Physiology and Chronic Health Evaluation II (APACHE II), Hematocrit (HCT), Hemoglobin (Hb), Platelet (PLT), Red Cell Distribution Width (RDW), Red Blood Cell (RBC), White Blood Cell (WBC), Albumin (ALB), Anion Gap (AG), Total Calcium (TCa), Chloride (Cl), Glucose (Glu), Potassium (K), Sodium (Na), Magnesium (Mg), Total Carbon Dioxide (TCO_2), Lactate (Lac), Partial Pressure of Carbon Dioxide (PCO_2), Potential of Hydrogen (Blood pH) (PH), Partial Pressure of Oxygen (PO_2), International Normalized Ratio (INR), Prothrombin Time (PT), Activated Partial Thromboplastin Time (APTT), Alanine Aminotransferase (ALT), Aspartate Aminotransferase (AST), Total Bilirubin (TB), Creatinine (CRE), Blood Urea Nitrogen (BUN), Lactate Dehydrogenase (LDH), Vasopressor Administration (Sa), Ventricular Pacing (VP), Glucocorticoid Use (GC).

Table S4: Coherent linear screening (VIF) in each cohort

ICU-28Dead		Hosp_28		external validation	
**Variable Names**	**VIF**	**Variable Names**	**VIF**	**Variable Names**	**VIF**
logPIV	1.55	logPIV	1.55	logPIV	1.60
Age	2.47	Age	2.38	SOFA	4.13
hypertension	1.58	hypertension	1.59	APSIII	4.93
AKI	1.36	AKI	1.38	SAPSII	3.50
CKD	2.01	CKD	2.17	OASIS	2.18
DM	1.46	DM	1.69	APACHEII	3.46
HF	1.89	HF	1.95	Spo2	1.28
COPD	1.26	COPD	1.29	RDW	1.71
SOFA	3.35	SOFA	3.30	WBC	1.52
APSIII	4.85	APSIII	5.04	ALB	1.23
SAPSII	4.47	SAPSII	5.25	AG	3.43
OASIS	2.65	OASIS	2.77	Cl	1.94
CCI	2.67	CCI	2.83	Lac	2.53
APACHEII	4.12	APACHEII	4.42	PCO2	3.30
RR	1.17	Spo2	1.20	PH	3.27
Spo2	1.19	RDW	1.73	INR	1487.18
temperaturef	1.10	WBC	1.66	PT	1485.57
RDW	1.61	ALB	1.32	APTT	1.57
RBC	8.12	AG	2.46	TB	2.50
WBC	1.68	Cl	1.42	AKI	1.51

ICU-28Dead		Hosp_28		external validation	
**Variable Names**	**VIF**	**Variable Names**	**VIF**	**Variable Names**	**VIF**
ALB	1.32	Glu	1.39	HF	1.44
AG	2.28	Lac	2.37	VP	1.40
Cl	1.43	PH	1.67		
Lac	2.11	PO2	1.28		
PH	1.54	APTT	1.26		
PO2	1.25	AST	1.59		
APTT	1.15	TB	2.04		
AST	1.39	CRE	2.51		
TB	2.11	BUN	2.52		
CRE	2.34	LDH	1.53		
BUN	2.25	CRRT	1.63		
LDH	1.39	Ventilation	1.26		
CRRT	1.60	Sa	1.60		
Ventilation	1.26	VP	1.55		
Sa	1.60	GC	1.22		
VP	1.54	PT	622.46		
GC	1.21	INR	621.46		
INR	222.95	HCT	25.96		
PT	221.55	Hb	26.07		
Hb	8.15	RR	1.22		

Note: Variance Inflation Factor (VIF) analysis for assessing multicollinearity among candidate predictor variables across the training cohort for two endpoints (ICU-28Dead, Hosp_28) and the external validation cohort. A VIF value greater than 5 was considered indicative of significant multicollinearity, and the corresponding variables were excluded from the respective final regression models to ensure model stability. Abbreviations: Acute Kidney Injury (AKI), Acute

Physiology and Chronic Health Evaluation II (APACHE II), Acute Physiology Score III (APS III), Activated Partial Thromboplastin Time (APTT), Alanine Aminotransferase (AST), Albumin (ALB), Anion Gap (AG), Aspartate Aminotransferase (AST), Blood Urea Nitrogen (BUN), Charlson Comorbidity Index (CCI), Chloride (Cl), Chronic Kidney Disease (CKD), Chronic Obstructive Pulmonary Disease (COPD), Continuous Renal Replacement Therapy (CRRT), Creatinine (CRE), Diabetes Mellitus (DM), Glucocorticoid Use (GC), Glucose (Glu), Heart Failure (HF), Hematocrit (HCT), Hemoglobin (Hb), International Normalized Ratio (INR), Intensive Care Unit (ICU), Lactate (Lac), Lactate Dehydrogenase (LDH), Log-transformed Pan-immune-inflammation Index (logPIV), Non-Invasive Blood Pressure—Diastolic (NBPD), Non-Invasive Blood Pressure—Mean (NBPM), Non-Invasive Blood Pressure—Systolic (NBPS), Oxford Acute Severity of Illness Score (OASIS), Partial Pressure of Carbon Dioxide (PCO_2), Partial Pressure of Oxygen (PO_2), Peripheral Oxygen Saturation (SpO_2), Platelet (PLT), Potassium (K), Prothrombin Time (PT), Red Blood Cell (RBC), Red Cell Distribution Width (RDW), Respiratory Rate (RR), Sequential Organ Failure Assessment (SOFA), Simplified Acute Physiology Score II (SAPS II), Sodium (Na), Total Bilirubin (TB), Total Calcium (TCa), Total Carbon Dioxide (TCO_2), Vasopressor Administration (Sa), Ventilation, Ventricular Pacing (VP), White Blood Cell (WBC).

Table S5: Incremental Prognostic Value of PIV Beyond Established Severity Scores (ICU 28-day Mortality)

Severity Score	AUROC (Base Model)	AUROC (Base + PIV)	ΔAUROC	P value for ΔAUROC	Category-based NRI (95% CI)	Continuous NRI (95% CI)	IDI (95% CI)
SOFA	0.6646	0.7139	0.0493	<0.001	0.2060 (0.1423–0.2683)	0.3969 (0.2581–0.5294)	0.0361 (0.0250–0.0472)
APSIII	0.5	0.634	0.134	<0.001	0.1341 (0.0712–0.1978)	0.3511 (0.2182–0.4859)	0.0303 (0.0218–0.0398)
SAPSII	0.5462	0.6441	0.0979	<0.001	0.1519 (0.0879–0.2144)	0.3507 (0.2150–0.4850)	0.0315 (0.0226–0.0414)
OASIS	0.6433	0.6851	0.0418	0.003	0.1140 (0.0481–0.1787)	0.3149 (0.1740–0.4489)	0.0250 (0.0157–0.0343)
CCI	0.6358	0.6898	0.054	<0.001	0.1353 (0.0636–0.2057)	0.3484 (0.2108–0.4777)	0.0317 (0.0224–0.0418)
APACHEII	0.6664	0.7022	0.0358	0.007	0.1366 (0.0736–0.2045)	0.3255 (0.1871–0.4605)	0.0285 (0.0182–0.0386)
